# Immobile lipopolysaccharides and outer membrane proteins differentially segregate in growing *Escherichia coli*

**DOI:** 10.1073/pnas.2414725122

**Published:** 2025-03-03

**Authors:** Sandip Kumar, Patrick G. Inns, Scott Ward, Valentine Lagage, Jingyu Wang, Renata Kaminska, Martin J. Booth, Stephan Uphoff, Edward A. K. Cohen, Gideon Mamou, Colin Kleanthous

**Affiliations:** ^a^Department of Biochemistry, University of Oxford, Oxford OX1 3QU, United Kingdom; ^b^Department of Mathematics, Imperial College London, London SW7 1AZ, United Kingdom; ^c^Department of Engineering Science, University of Oxford, Oxford OX1 3PJ, United Kingdom

**Keywords:** gram-negative bacteria, bacterial envelope, lipopolysaccharides, membrane biogeneis, outer-membrane proteins

## Abstract

The outer membrane (OM) of gram-negative bacteria is a robust, impermeable barrier that excludes many classes of antibiotics. Contrary to the classical model of an asymmetric lipid bilayer, recent evidence suggests the OM is predominantly an asymmetric proteolipid membrane (APLM). Outer leaflet lipopolysaccharides (LPS) that surround integral β-barrel outer membrane proteins (OMPs) are shared with other OMPs to form a supramolecular network in which the levels of OMPs approach those of LPS. Some of the most abundant OMPs in the *Escherichia coli* OM are trimeric porins. How porins and LPS are incorporated into the OM of growing bacteria is poorly understood. Here, we use live-cell imaging and microfluidics to investigate how LPS, labeled using click chemistry, and the porin OmpF, labeled using the bacteriocin colicin N, are incorporated into the *E. coli* OM. Diffraction-limited fluorescence microscopy shows OmpF and LPS to be uniformly distributed and immobile. However, clustering of both macromolecules becomes evident by superresolution microscopy, which is also the case for their biogenesis proteins, BamA and LptD, respectively. Notwithstanding these common organizational features, OmpF insertion into the OM is cell-cycle-dependent leading to binary partitioning and strong polar accumulation of old OmpF. Old LPS on the other hand is diluted ~50% at each division cycle by new LPS, resulting in only mild polar accumulation of preexisting LPS. We conclude that although LPS and OMPs are destined to form the APLM their insertion dynamics are fundamentally different, which has major implications for understanding how the OM is assembled.

## Significance

The rise in antibiotic resistance among gram-negative bacteria is due in part to the impermeable outer membrane (OM). Classically described as an asymmetric lipid membrane, recent evidence suggests the OM is predominantly an asymmetric proteolipid membrane (APLM). Here, we demonstrate that while the major porins and lipopolysaccharides (LPS) which dominate APLM networks are similarly deposited as clusters by clustered arrangements of biogenesis machines in the *Escherichia coli* OM, their insertion dynamics differ. Porin biogenesis is mid-cell biased while that of LPSis largely unrestricted. These alternate insertion strategies offer clues as to how the impermeable APLM is assembled in living bacteria.

The outer membrane (OM) of gram-negative bacteria is a complex barrier that excludes large (>600 Da) polar and nonpolar molecules (1). The OM’s impermeability is a major factor in the global rise of antibiotic resistance among organisms such as *Escherichia coli*, *Pseudomonas aeruginosa,* and *Klebsiella pneumoniae*, which are leading causes of pneumonia and sepsis (2). Consequently, targeting OM biogenesis with drugs that disrupt its assembly has the potential to identify new classes of antibiotics specific for gram-negative bacteria (3, 4). The defining characteristic of the OM is its lipid asymmetry, first described by Nikaido and colleagues (5, 6), in which the outer leaflet is composed of lipopolysaccharides (LPS) and the inner leaflet of phospholipids (PLs). Immersed in this asymmetric bilayer are integral β-barrel outer membrane proteins (OMPs) which are built from even-numbered β-strands (8 to 36) and are typically monomeric, dimeric, or trimeric. OMPs carry out the many functions of the OM, which include assembling the membrane (7, 8), forming stabilizing contacts with the underlying peptidoglycan (PG) cell wall (9), acting as conduits for exchange of metabolites with the environment (1), actively importing essential nutrients such as metal chelate complexes and glycans (10), adhering to host epithelia during pathogenesis (11), degrading antimicrobial peptides (12), and mediating intercellular communication and expelling antibiotics (13).

The impermeability and semicrystalline nature of the OM has traditionally been attributed to its asymmetric lipids (14–18). By contrast, OMPs have generally been regarded as incidental to the biophysical properties of the OM. This is a reasonable assumption given the variable abundances (10^2^–10^5^ protein copies/cell) and sizes (10 to >100 kDa) of individual OMPs and the varying nature of the OM proteome, which typically comprises dozens of different proteins. A series of recent advances however suggest that OMPs are integral to OM stability due to the formation of a spatiotemporally organized superstructure: 1) Total internal reflection fluorescence (TIRF) microscopy of TonB-dependent transporters (TBDTs) in *E. coli* demonstrates that these monomeric OMPs form islands or clusters in the membrane that are shunted to the poles as cells expand their OM, leading to binary partitioning following division (19). Similar OMP clustering and polar migration has been observed for the substrate specific porin LamB (20). Since the OM is not energized and there is no ATP in the periplasm for active protein degradation, binary partitioning enables bacteria to passively exchange their OMPs in response to changes in the environment. 2) The insertion of TBDTs into the OM is dependent on the cell cycle, whereby maximal biogenesis occurs at division septa and little or no biogenesis occurs at cell poles. This cell cycle dependence is governed by the maturation state of the cell wall which regulates the OMP insertion activity of BamA. New PG at division sites is a poor inhibitor of BamA while old PG at cell poles suppresses BamA activity. As a result, OM and PG biogenesis mirror each other (21), adding to the growing body of evidence that cell envelope biogenesis is a highly coordinated process (22). The control of BamA by PG, coupled with the inability of OMPs to diffuse laterally in the OM, accounts for the spatiotemporal behavior of OMPs and leads to binary partitioning. 3) Atomic force microscopy of live *E. coli* cells shows that the OM is phase-separated into OMP-rich and LPS-rich regions and that OMP-rich regions dominate the surface (23). 4) Photoactivatable crosslinking from the β-barrels of OMPs into the membrane shows that individual OMPs are enveloped by asymmetric lipids (24). These lipids, particularly LPS in the outer leaflet, mediate promiscuous associations with other OMPs. Based on inter-OMP distance and symmetry restraints derived from live cell AFM, coarse-grain modeling suggests that supramolecular OMP assembly stems from imperfect hexagonal arrangements of heterologous OMPs bound together by interfacial LPS (24). 5) Similar six-fold symmetry has been observed by AFM for flattened OM vesicles treated with polymyxin antibiotics that bind LPS (25). However, it remains to be established whether these symmetrical arrangements are due to preorganized LPS within OMP clusters or to polymyxins driving LPS organization in the absence of OMPs (26, 27). 6) Depletion of OMPs from the OM results in alterations in cell shape, infiltration of phospholipids into the outer leaflet, and destabilization of the membrane, changes that are consistent with a structural role for OMPs that goes beyond their individual biological function (28).

Collectively, these observations support a supramolecular model for the OM where both OMPs and LPS contribute to its structural organization, stability, and load-bearing capacity (29). The insertion of OMPs and LPS into the OM by the β-barrel assembly machinery (BAM) and the lipopolysaccharide transport (Lpt) machinery, respectively, have been characterized extensively (7, 8). Yet we know little of these processes in live bacteria. Here, we address this problem using fluorescently labeled OmpF, an abundant OMP, and LPS using diffraction-limited and subdiffraction imaging, which demonstrate that both cluster in the *E. coli* OM. We also find that although these molecules are destined for the same supramolecular network they exhibit different insertion strategies, which leads to differential segregation as bacteria grow and divide. We propose these differences point to distinct cellular-level control of LPS incorporation into the OM compared to that of OMPs which, along with the clustered insertion of all OM components, has ramifications as to how the APLM is assembled in a growing bacterium.

## Results

It has been estimated that up to half the mass of the OM is accounted for by OMPs (30). However, the OMPs that have thus far served as indicators of *E. coli* OMP organization in fluorescence microscopy experiments are low abundance TBDTs, which are typically present at <2,000 copies per cell (31). By contrast, OMP networks are dominated by high abundance porins such as OmpF (23), which proteomics studies suggest is present at ~70,000-80,000 copies per cell. By comparison, >10^6^ LPS molecules are estimated to be present in the outer leaflet of the OM (32). Here, we investigate the surface distribution and behavior of fluorescently labeled OmpF and LPS in growing bacteria that together form the major APLM networks of the *E. coli* OM.

### OmpF and LPS Cluster in the OM.

**Fig. 1.**
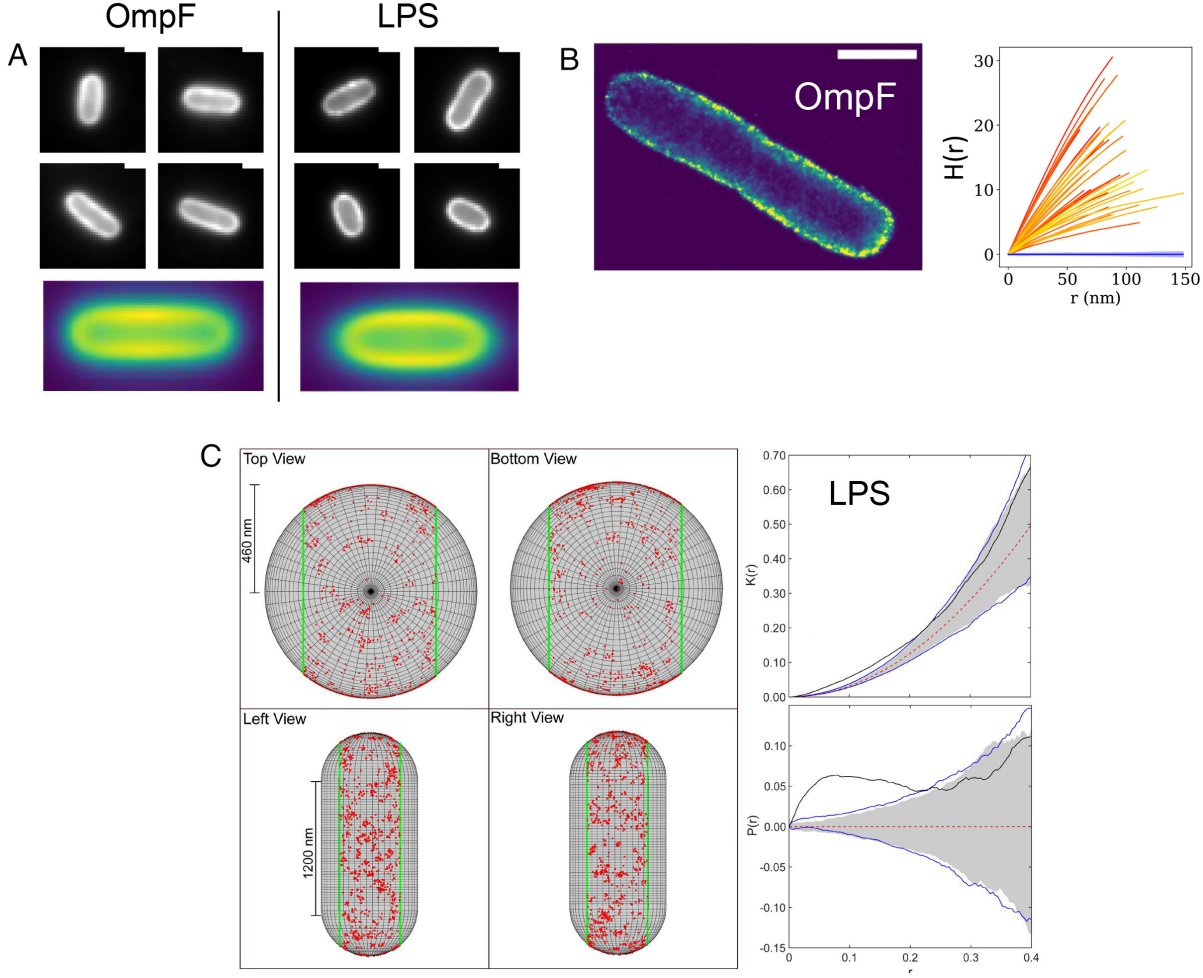
Clusters of OmpF and LPS envelope the outer membrane (OM) of *E. coli* MG1655. (*A*) Selection of images for OmpF, labeled with Colicin N^1-185^mCherry, and LPS, labeled by Kdo-azide and Alexa Fluor-488 sDIBO, showing that by epifluorescence cells are completely coated by each molecule. Space bars throughout, 1 μm. Below each selection are heatmaps for the distribution of fluorescence from each channel where fluorescence intensity was normalized between 0 and 1, the long axis normalized between 0 and 100, and the short axis normalized between 0 and 50 for multiple cells; n = 3,931 cells for OmpF and n = 4,025 cells for LPS. (*B*) Representative image of 2D-PALM data for *E. coli* MG1655 OmpF labeled with colicin N^1-185^PAmCherry (*Left* panel) and Ripley’s H-function spatial statistics output for 2D-PALM data for 35 cells (*orange*) compared to 9999 Complete Spatial Randomness (CSR) simulations (*light blue*) fitted to a Poisson distribution (*dark blue line*). In every case, the experimental data for OmpF escape the CSR simulations, implying clustering in the OM. Accompanying spatial statistics functions (Ripley’s K and L) are shown in *SI Appendix*, Fig. S2*B*. (*C*) Global analysis of LPS localizations in a stationary phase *E. coli* MG1655 cell obtained from 4Pi-Single Molecule Switching (4Pi-SMS) superresolution microscopy (39). *Right* panel show LPS localizations mapped onto a cylinder and one pole with the dimensions of an average *E. coli* cell. *Top Left* panel shows the plot for the observed inhomogeneous K-function (*black line*), with the theoretical value (*dashed red line*) and CSR simulation envelopes (*gray*), and double blink model (*blue lines*); an insert of the raw data is shown in raw data inset. *Bottom Left* panel shows the observed inhomogeneous P-function (*black line*), with the theoretical value (*dashed red line*), simulation envelopes for the CSR simulations (*gray*), and the double blink model (*blue lines*). LPS distributions were analyzed either globally, as shown in this panel, or piecewise where the cell cylinder and caps were analyzed separately. Three stationary phase cells were analyzed and two exponential phase cells were analyzed by one or both of these approaches. All exhibited clustered LPS distributions (*SI Appendix*, Figs. S3–S11).

Before exploring the effects of growth and division on populations of LPS and OmpF, we investigated the surface distribution of these abundant molecules by diffraction-limited and superresolution fluorescence microscopy. LPS was labeled covalently by incorporation of Kdo-azide into *E. coli* MG1655 rough LPS, which lacks O-antigen (33). Copper-free click chemistry (34) was then used to conjugate AlexaFluor488 or AlexaFluor647 dyes into surface-exposed LPS (*Materials and Methods*). OmpF was labeled noncovalently using high-affinity GFP, mCherry, or PAmCherry fusions of colicin N (ColN^1-185^-GFP, ColN^1-185^-mCherry, and ColN^1-185^-PAmCherry, respectively) (*Materials and Methods* and ref. 35). Each of the LPS^AF488^ and OmpF^ColN1-185^-mCherry labels displayed essentially uniform distribution when imaged by epifluorescence microscopy, in agreement with previous studies (35, 36) (Fig. 1*A*). Moreover, fluorescence recovery after photobleaching (FRAP) experiments with each label demonstrated no recovery of fluorescence in a two-minute period (*SI Appendix*, Fig. S1). The immobility of OMPs has been reported a number of times (19, 35), which is replicated here. However, LPS mobility has proven controversial, some studies suggest immobile LPS (37) while others suggest LPS diffusion in the OM (38). Our data demonstrate that both LPS and OMPs are immobile once inserted into the OM.

We next explored the surface distribution of OmpF and LPS at subdiffraction resolution in live *E. coli* MG1655 cells. Initially, we focused on OmpF which was labeled with ColN^1-185^-PAmCherry and its OM localization determined by 2D-photoactivated localization TIRF microscopy (2D-PALM-TIRFM). To ascertain from these 2D data if OmpF is randomly distributed in the OM or forms clusters, we implemented spatial summary statistical analysis known as Ripley’s K function in which data on xy coordinates are compared to completely spatially random (CSR) simulated datasets (*Materials and Methods*) (Fig. 1*B*). For each of the 35 cells analyzed, the experimental traces escaped the envelopes of 2D CSR simulations in a positive direction, implying clustering of OmpF trimers in the OM (Fig. 1*B* and Movie S1). *E. coli* MG1655 expresses both major porins, OmpF and OmpC. The porins share ~60% sequence identity and form hybrid trimers in the OM (40). ColN^1-185^-PAmCherry does not bind OmpC (35) and so we repeated the 2D-PALM-TIRFM experiments and cluster analysis using an *ompC* deletion strain (BE3000), which confirmed clustering of OmpF even in the absence of its close homologue OmpC (*SI Appendix*, Fig. S2).

Determining the surface distribution of LPS in the OM of live *E. coli* cells by fluorescence microscopy presented a major technical challenge. To address this problem, we used AF^647^-labeled LPS *E. coli* MG1655 cells in 3D-Stochastic Optical Reconstruction Microscopy (3D-dSTORM) to localize individual LPS molecules and analyzed the data using newly developed spatial statistical methods (41) that take account of the cylindrical shape of *E. coli* (Fig. 1*C*). Details of the mathematics underpinning the 3D analysis of LPS distributions are presented in *SI Appendix*. Click-based labeling of LPS required extended (overnight) labeling when cells are in stationary phase. We analyzed LPS distributions by 3D-dSTORM for both stationary- and exponential-phase cells, the latter following a brief period of growth in fresh media (*SI Appendix*, Figs. S3–S11). Experimental data escaped the envelopes of CSR simulations in a positive direction for both types of cells indicating that LPS clustering is evident in stationary and exponential phase cells. LPS cluster size was estimated from these data to be in the range 25 to 50 nm which is similar to that reported by AFM for LPS patches (23).

Finally, we imaged the biogenesis OMPs BamA and LptD, labeled with monoclonal antibodies (21, 24), using 3D-structured illumination microscopy (3D-SIM). Both OMPs were organized as islands that were randomly distributed in the OM (*SI Appendix*, Fig. S12). Clusters of BamA have previously been reported in both fixed and live cells (21, 42). In summary, OmpF and LPS, two major components of the *E. coli* APLM, exist in the form of clusters. Since OMPs and LPS cannot diffuse laterally, this observation implies they are deposited in the OM as clusters by their biogenesis OMPs BamA and LptD, which are also organized as clusters in the OM.

### LPS and OmpF Differentially Segregate in Dividing Cells.

While subdiffraction imaging highlighted the widely distributed but clustered nature of OmpF and LPS and FRAP showed that both are immobile in the OM, these approaches revealed little about their behavior in growing and dividing cells. We therefore sought to compare the distribution of labeled LPS and OmpF as cells extend their cell envelopes and proceed through division. Initially, we looked at OMP biogenesis which previous studies on low abundance TBDTs have shown is linked to the cell cycle through PG control of BamA OMP insertion activity (19, 21). We found that the emergence of OmpF on the surface of *E. coli* MG1655 and the porin-deficient strain BZB1107, the latter via plasmid-based expression, exhibited similar cell-cycle dependence as that of low-abundance OMPS (*SI Appendix*, Fig. S13).

**Fig. 2.**
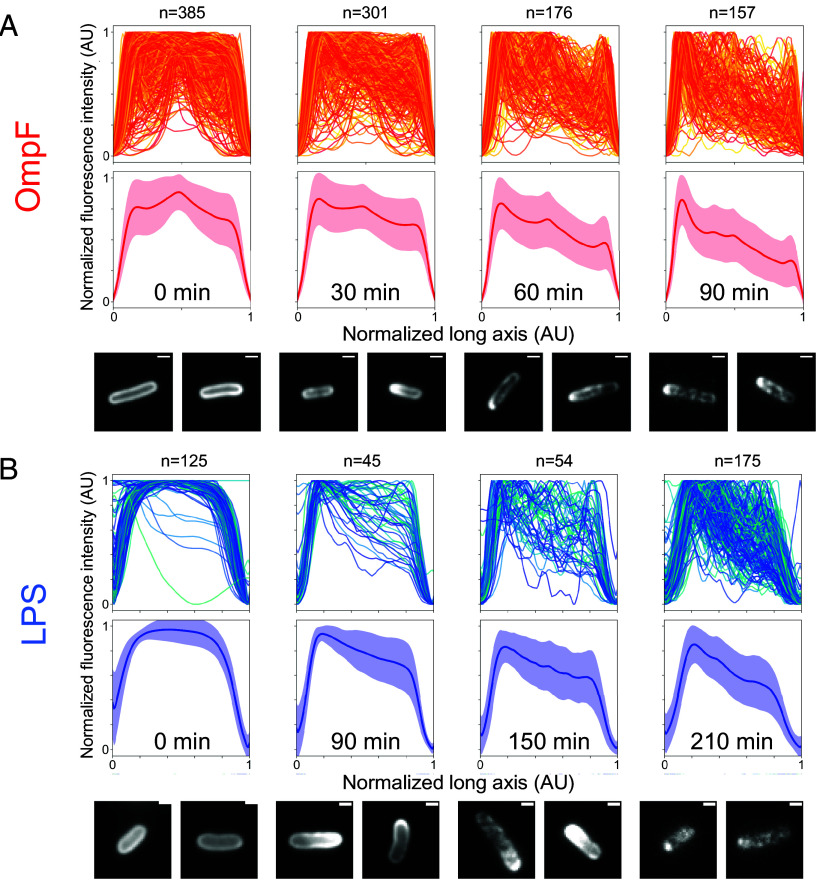
Pulse–chase experiments show growth-driven polar migration of OmpF and LPS. (*A*) *E. coli* cells were labeled using Colicin N^1-185^ mCherry at t = 0 min (*Materials and Methods*). Cells were then resuspended in fresh media for further growth and samples collected at the indicated time points. *Upper* panels, Normalized fluorescence intensity profiles along the long axis of cells. *Middle* panels, average normalized fluorescence intensity profiles. *Red line*, mean fluorescence intensity profile. *Pink envelope*, (±) SD. *Lower* panels, Representative cell images from each time point. All images were contrast adjusted to the same arbitrary value for visibility. (Scale bar, 1 μm.) (*B*) *E. coli* LPS was labeled using Alexa Fluor-488 sDIBO at t = 0 min (*Materials and Methods*). Cells were then resuspended in fresh media for further growth and samples collected at the indicated time points. *Upper* panels, Normalized fluorescence intensity profiles along the long axis of cells. *Middle* panels, average normalized fluorescence intensity profile. *Blue line*, mean fluorescence intensity profile. *Light blue envelope*, (±) SD. *Lower* panels, Representative cell images from each time point. All images were contrast adjusted to the same arbitrary value for visibility. (Scale bar, 1 μm.)

**Fig. 3.**
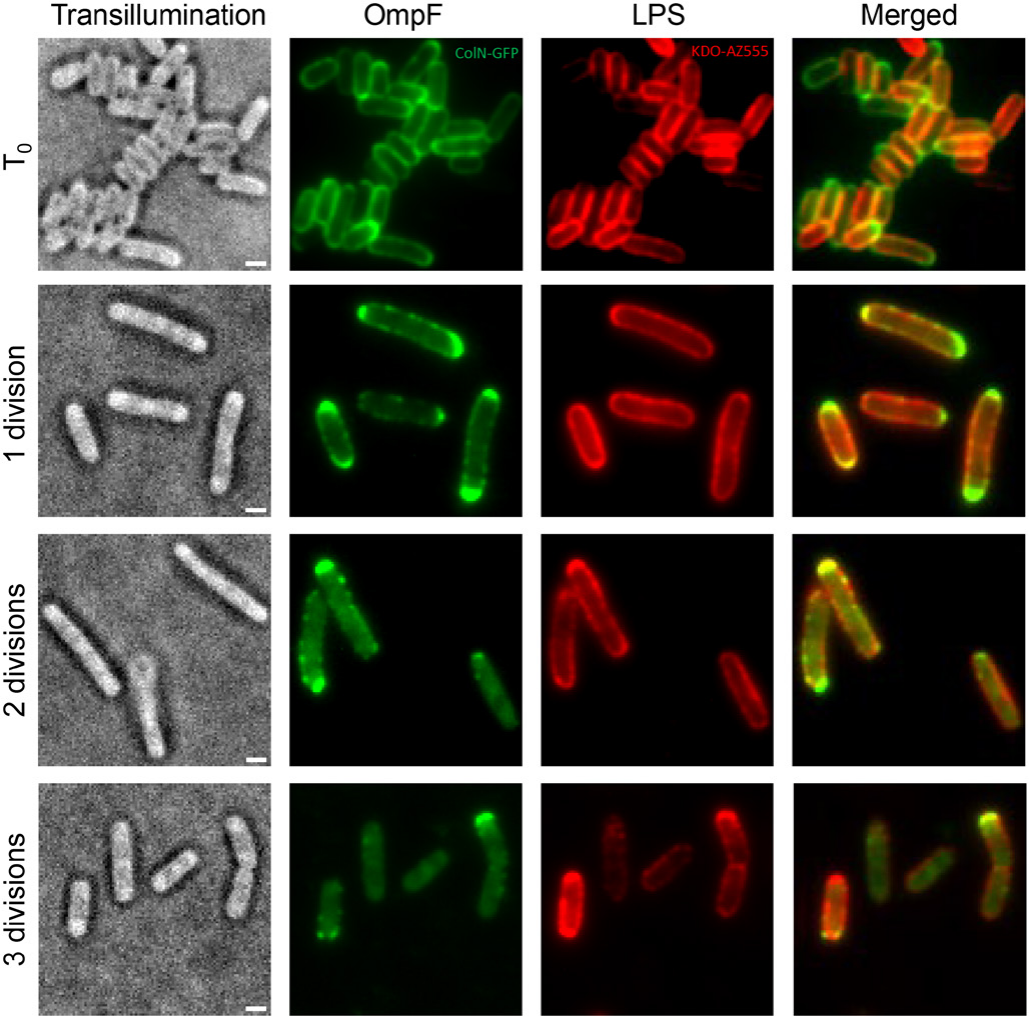
Divergent spatiotemporal growth dynamics of OmpF and LPS. *E. coli* MG1655 cells were colabeled in stationary phase using KDO-azide and Alexa Fluor-555 for LPS and ColN-GFP for OmpF. Cells were subsequently revived in fresh LB and imaged by TIRFM on agar pads at the indicated time points. Division times were calculated based on the OD_600_ of the culture following the resuspension in fresh media. (Scale bar, 1 μm.)

Since OMPs and LPS together form the APLM (24), we hypothesized that LPS would undergo binary partitioning like that of OMPs (19). We tested this hypothesis in two ways. First, we compared the rate of polar displacement of LPS and OmpF separately in *E. coli* pulse–chase experiments, where populations of cells were pulse-labeled for each macromolecule and followed over several rounds of division. Normalized fluorescence intensity profiles were then compared for multiple cells at given time points following alignment of cells at their most fluorescent pole (Fig. 2). Surprisingly, the apparent rate of polar displacement of labeled (old) LPS as a result of growth and division was two-to-three-fold slower than that of OmpF. Second, we colabeled LPS and OmpF in the same *E. coli* cells and followed their distribution following multiple rounds of division. We found that immediately after labeling both molecules were largely uniformly distributed across the long axis of cells (Fig. 3). After one doubling time almost the entirety of labeled (old) OmpF localized to the poles whereas labeled LPS distribution remained close to uniform. Following two doubling times, labeled OmpF was present at a single pole of every cell while LPS distribution had begun to be biased to one pole. Finally, after approximately three doubling times (*SI Appendix*, Fig. S14), the distribution of old LPS became moderately polar whereas old OmpF was heterogeneously distributed among cells in the population (Fig. 3), which is a natural consequence of binary OMP partitioning where some daughter cells do not retain any of the original labeled protein (19).

Our experiments show that the incorporation of high abundance trimeric porins into the OM is cell cycle dependent, similar to that reported previously for low abundance TBDTs. Once inserted, porins and LPS migrate at different apparent rates toward the poles as *E. coli* cells grow and divide even though both molecules participate in the APLM. The net result is differential segregation within individual bacterial cells.

### LPS Is Continuously Inserted Across the OM.

**Fig. 4.**
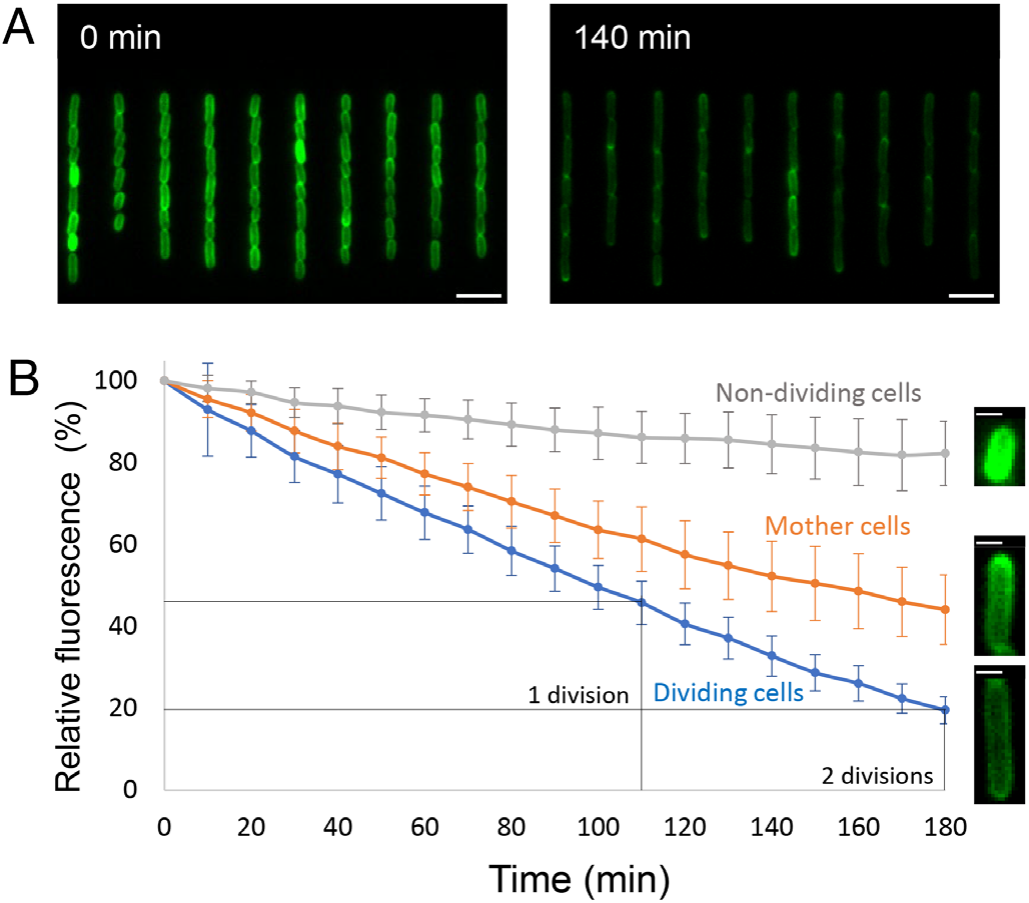
Labeled LPS is diluted by the addition of unlabeled LPS as *E. coli* cells grow. Continuous tracking of LPS distribution in microfluidic flow channels (Mother Machine) for growing *E. coli* cells pulse-labeled with KDO-azide and Alexa Fluor-488 sDIBO. See also *SI Appendix*, Fig. S15. (*A*) Representative images of cells at the indicated time points post labeling. (Scale bar, 5 μm.) (*B*) Decay of the relative fluorescence intensity in dividing, nondividing, and mother cells and a representative image of each group after 180 min. Division time was based on the rate of extension and division of multiple cells. n_dividing_ = 600 cells, n_mother_ = 85 cells, n_nondividing_ = 35 cells. Error bars represent the SD for each group and time point. (Scale bar, 1 μm.)

The difference in OmpF and LPS growth dynamics suggested LPS insertion into the *E. coli* OM is more uniform than that of OMPs. To test this hypothesis, we continuously tracked the migration of old LPS during cell growth using a microfluidic setup known as the Mother Machine (43, 44). The LPS of *E. coli* cells was prelabeled as described above, loaded into microfluidic flow-chambers and fresh growth media immediately introduced under flow. Tracking the relative fluorescence of cells over 3 h of growth in the channels (equivalent to 2 to 3 division cycles under these conditions) showed constant dilution of the fluorescent signal as the OM extended and cells divided (Fig. 4*A* and *SI Appendix*, Fig. S15 and Movie S2). For most cells, the rate of fluorescence reduction was proportional to their extension (Fig. 4*B* and *SI Appendix*, Fig. S15*B*), indicating that preexisting LPS is essentially being diluted by the addition of new, unlabeled LPS. Interestingly, some cells failed to grow, their levels of uniform fluorescence remaining essentially unchanged after 3 h (Fig. 4*B*). These growth outliers, which we assume to be dead cells, were used as internal fluorescence controls against which other cells in the same Mother Machine experiment were compared directly. The constant level of fluorescence in these nongrowing cells implies that the changing signal across all cells is unlikely to be the result of photobleaching or the label being washed out or lost due to shear forces within the microfluidic flow chamber. Our experiments also revealed a third type of LPS dynamics, observed predominantly in mother cells at the closed end of each growth channel furthest from the media flow channel, but also in some daughter cells further up the flow channel (*SI Appendix*, Fig. S15*A*). The decay of fluorescence signal in these cells was not proportional to their extension and the decay was slower than that of new daughter cells. Closer examination of these older cells over time showed that they have moderate accumulation of old LPS at their poles, which explains their reduced fluorescence signal decay rate (Fig. 4*B* and Movie S2). Nevertheless, the growth rate of these cells was similar to that of new daughter cells (*SI Appendix*, Fig. S16 and Movie S3). We conclude that the continuous insertion of LPS into the OM leads to simple dilution of old LPS molecules, with a slow accumulation of preexisting LPS occurring in the oldest poles of progenitor cells.

## Discussion

Contrary to accepted dogma, the *E. coli* OM is predominantly an asymmetric proteolipid membrane (APLM) where OMPs and LPS come together to form networks with imperfect hexagonal symmetry that are interspersed with LPS-only regions or patches. Here, we provide evidence supporting elements of this model, furnish insights as to how component parts of the APLM are incorporated into the OM and suggest how the resulting network might be assembled in growing bacteria while ensuring its impermeability.

Previous work has shown that low abundance TBDTs, BtuB, Cir, and FepA, exist as puncta/clusters/islands in the *E. coli* OM where multiple copies are located (19, 21). In some instances, the copy number of specific OMPs within individual clusters has been estimated from photobleaching analysis, for example 20 to 30 protein copies of BtuB and FepA reside within specifically labeled islands (24). Here, 3D-SIM visualized the clustered organization of BamA and LptD and demonstrated their distribution throughout the OM. Moreover, superresolution fluorescence microscopy showed that clustering is also evident for porins and LPS. Combined with past reports, the present work suggests that all OMPs, their biogenesis proteins, and LPS exist in the OM in the form of clusters. This leads us to conclude that the OM is assembled from these clusters, which presumably retain their clustered form due to the twin effects of their mode of insertion into the OM and corralling by the immobile APLM.

Our work shows that control of porin and LPS insertion into the OM is fundamentally different in *E. coli*. The incorporation of porins is cell cycle dependent wherein the major site of insertion is the septum, with lesser incorporation on the long axis. Thereafter, cell elongation followed by division leads to binary partitioning of the OMP, as seen with low-abundance OMPs. In contrast, LPS insertion is widespread and constitutive, leading to preexisting LPS being diluted as cells grow. Hence, the maturation state of the cell wall which is the master regulator of OMP insertion by BamA in the OM (21) does not similarly influence LPS insertion by the LptDE complex. Instead, the protein bridges of the Lpt system that pass through the cell wall must continually insert LPS into the OM (see below). The exceptions to this even incorporation of LPS across the surface are the cell poles. We speculate that the slow accumulation of LPS at the poles may be indicative of new LPS insertion being less energetically favorable due to cell curvature. We note that LPS insertion across the OM of *E. coli*, a γ-proteobacterium, is distinct from that recently described for the unipolar growth of the α-proteobacterium *Brucella abortus*. LPS insertion is localized to the new pole in *B. abortus*, which colocalizes with the biogenesis of new OMPs and PG (45, 46).

**Fig. 5.**
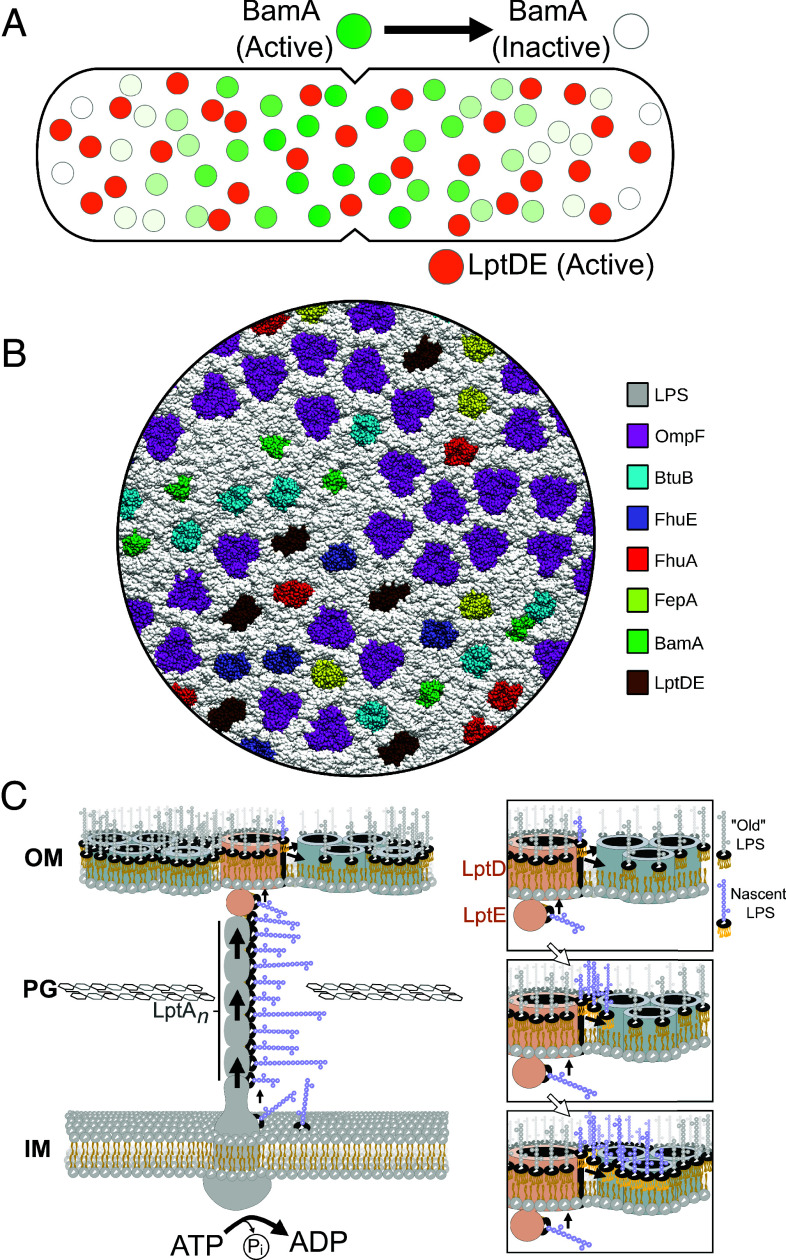
Models depicting assembly of the *E. coli* asymmetric proteolipid membrane (APLM). (*A*) Clusters of biogenesis proteins BamA and LptD (along with their accessory lipoproteins) are randomly distributed throughout the OM. Image shows a dividing cell where BamA clusters (*green*), are most active for OMP biogenesis at the division plane but largely inactive at cell poles. LptD clusters by contrast (*orange*) are constitutively active for LPS insertion throughout the OM. (*B*) Coarse-grain model of a simulated APLM where OMP–OMP associations are mediated by interfacial LPS (*gray*). Figure adapted from (24). The intrinsic hexagonal symmetry of the APLM centers on clusters of OmpF (*pink*). BamA (*green*) and LptD (*brown*), as well as TBDTs (BtuB, *cyan*; FhuA, *red*; FepA, *yellow*; FhuE, *mauve*), colocate with OmpF within these biogenesis clusters (24). See text for details. (*C*) LPS secretion as a driving force for remodeling the OM. Main panel shows the ATP-dependent deposition of LPS into the OM by the Lpt system which we suggest could overcome the inherent immobility of OMPs and LPS to form the APLM (*Discussion*). *Right* panels, continual LPS deposition pushes further into the membrane to envelope the β-barrel of an OMP and seal the membrane.

A key question posed by the APLM model is the relationship between OMP and LPS clusters/islands observed by fluorescence microscopy (19) and the proteolipid network observed by AFM (23). While much remains to be discovered about this relationship, we hypothesize the following scenarios. Clusters of BamA (and their associated lipoproteins BamBCDE) and LptDE randomly deposit their substrates, OMPs and LPS, respectively, into the OM. Distribution of the biogenesis machines throughout the OM ensures the APLM covers the cell (Fig. 5*A*). Whether the resulting network has the same lattice structure pole-to-pole, given the incongruous insertion strategies of OMPs and LPS, is not known. Assuming BamA does not discriminate between OMPs and the OM proteome simply reflects *omp* genes expressed under a given set of conditions, OMPs of the APLM share their enveloping LPS with other OMPs regardless of their abundance or function. Hence, both high abundance OMPs like OmpF become coresident with low abundance OMPs such as TBDTs as well as biogenesis OMPs themselves. AFM and photoactivatable crosslinking support the formation of such heterogeneous assemblies. AFM has demonstrated the close proximity of the TBDT FepA within networks of OmpF while crosslinking has shown that OmpF shares LPS with various TBDTs (FepA, FhuA, FhuE) as well as with LptD, and that BtuB shares LPS with BamA (Fig. 5*B*) (24). Finally, LPS-only patches observed by AFM (23), which essentially conform to the asymmetric membrane bilayer originally proposed by Nikaido, likely form when LptDE clusters are not close enough to BamA clusters to assemble the APLM. In these instances, the deposited LPS self-associates to produce asymmetric pieces of OM devoid of OMPs, which may correspond to the LPS clusters observed here by 3D-dSTORM.

Another important question posed by the APLM model is how an expanding OM can be assembled in growing bacteria when OMPs and LPS are unable to diffuse laterally in the membrane? The challenge this poses for maintaining OM impermeability is highlighted by recent coarse-grain simulations. As spaces were artificially introduced into a computational APLM to probe how the OM responds to restructuring, the limited mobility of its component parts resulted in poor packing that left gaps, some the size of the antibiotic vancomycin that is normally excluded from the OM (24). A possible solution to this assembly problem is the active insertion of LPS into the OM. Unlike OMPs, the insertion of which is energy-independent, LPS insertion into the OM by the Lpt system is driven by ATP hydrolysis in the cytoplasm (7). LPS is synthesized at and extracted from the inner membrane, shunted along a protein bridge through the periplasm, and finally deposited in the outer leaflet of the OM by the assembled LptB_2_FGCADE complex in an ATP-dependent manner (47). LPS loading stimulates the ATPase of the LptB_2_FG transporter and facilitates formation of the Lpt bridge. In addition, crosstalk between the LptDE translocon in the OM that inserts LPS and the inner membrane components is governed by the LPS status of the LptCA bridge (48). We speculate that another consequence of ATP-driven LPS secretion could be to push already deposited LPS and possibly even OMPs further into the membrane. Since LPS insertion occurs over much of the cell surface, active deposition could overcome the inherent immobility of the OM, simultaneously filling in gaps and rendering the APLM impermeable as the OM expands (Fig. 5*C*). Viewed in this context, LPS insertion is analogous to mortar being injected between tiles to make a stable and water-tight mosaic except in this instance the mortar may also be moving the tiles before the whole structure eventually rigidifies.

## Materials and Methods

### Strains, Plasmids, Oligonucleotides, and Antibodies used in this Study.

Bacterial strains used in this study are listed in *SI Appendix*, Table S1, plasmids and oligonucleotides are listed in *SI Appendix*, Tables S2 and S3, respectively, and antibodies and engineered bacteriocins are listed in *SI Appendix*, Table S4.

### Construction of pKBJ51 and pPGI1/3 Plasmids.

Construction of pKBJ51 has been described previously (35). In brief, the gene for ColN^1-185^-mCherry was ordered from GenScript with a 13-amino acid linker containing a tobacco etch virus protease site and 5’ NdeI and 3’ XhoI sites that allowed for digestion and ligation into a NdeI/XhoI pET21a vector, generating pKBJ51. Construction of pPGI1 and pPGI3 was carried out by amplifying the gene for GFP and PAmCherry from pNP4 and pROD85, respectively. 5’ BamHI site and a 3’ XhoI site were introduced using the primers detailed in *SI Appendix*, Table S3. For the digestion of this gene fragment and its vector pKBJ51, double restriction enzyme digests were conducted with single units of BamHI and XhoI in NEB CutSmart buffer. These reactions were conducted at 37 °C for 60 min. Subsequent DNA ligation was performed using NEB T4 ligase, the ratio of vector to insert was varied to maximize the likelihood of success.

### Expression and Purification of Antibodies and Bacteriocins.

#### αLptD-AF488 Fabs.

The expression and production of αLptD Fabs were described previously (49) and the use of αLptD-AF488 for labeling intact *E. coli* cells was carried as in (24).

#### αBamA-MAB2.

Anti-BamA MAB2 antibodies expression and production were previously described (50) and the use of αBamA-AF488 for labeling intact *E. coli* cells was carried as in (21).

#### ColN^1-185^ fusion proteins.

All C-terminal fluorescent fusions of ColN^1-185^ were expressed using *E. coli* BL21 (DE3). Cells were grown to an optical density of 0.5 to 0.7, and ColN expression was then induced with 1 mM IPTG for 3 to 4 h. Cells were then pelleted by centrifugation at 5,000 g for 12 min at room temperature. The supernatant was discarded and the pellet resuspended in a solution of 5 mM imidazole, 500 mM NaCl, 20 mM Tris-HCl, pH 7.5, treated with PMSF (1 mM), and cells then lysed by sonication. Cell debris was pelleted by centrifugation (12,500 RPM, 4 °C, 30 min) and the supernatant passed through a 220 nm filter. All Colicin N–based fluorescent labels harbored a C-terminal His6 tag. The first step in the purification was nickel affinity chromatography. Two GE HisTrap columns were connected in parallel. Filtered protein sample was loaded onto the column by either direct syringe injection or by superloop. Then, 5 mM imidazole, 500 mM NaCl, and 20 mM Tris-HCl, pH 7.5, was flowed through the columns to elute unbound protein. An increasing gradient of 750 mM imidazole, 500 mM NaCl, and 20 mM Tris-HCl, pH 7.5, was used for elution. Fractions were collected, analyzed by SDS-PAGE, pooled fractions treated with 5 mM EDTA and concentrated to <20 ml. The concentrated sample was dialyzed in 4 L, 25 mM Tris, and 150 mM NaCl, pH 7.5, at 4 °C, overnight (dialysis membrane: 12,000 to 14,000 MWCO), filtered, and fractionated by size exclusion chromatography using a Superdex S200 26/60 column eluted with 25 mM Tris and 150 mM NaCl, pH 7.5. Collected fractions were analyzed by SDS-PAGE, pooled, and stored at −20 °C.

### Induction of OmpF Expression for Surface Labeling.

Induction of OmpF expression was carried out as described previously (21). In brief, *E. coli* BZB1107 (Δ*ompF*, Δ*ompC*, Δ*lamB*) was transformed with a pBAD-HismycB plasmid encoding OmpF (pNGH71) and grown to mid-log phase. The culture was then induced with 1% arabinose for short bursts of 5, 7.5, or 10 min and fixed immediately with 4% formaldehyde for 30 min. Labeling was conducted after fixation with 200 nM ColicinN^1-185^mCherry for 1 h. Epifluorescence images of OmpF distribution were captured at each induction time. From these images, kymographs were generated to measure the change of fluorescence distribution with respect to cell length and the average fluorescence intensity of each cell at each induction time was determined and plotted.

### Bacterial Growth and Labeling for Microscopy.

#### OmpF and LptD imaging cell preparation.

For all strains, a day prior to microscopy a single colony of the target strain was grown in 10 ml LB at 37 °C, shaking. 500 µl of culture was pelleted and cells resuspended in M9-glucose (M9G) or M9G+Casamino acids (M9GC) to grow overnight at 37 °C, shaking. On the following day 50 to 500 µl of culture was pelleted and cells resuspended in 4 ml M9G or M9GC and grown to the required OD_600_.

#### LPS imaging cell preparation.

A single colony of the selected *E. coli* strain was grown overnight at 37˚C in LB broth while shaking. The overnight culture was then diluted to an OD_600_ of 0.05 in LB containing 1 mM KDO-azide (Click Chemistry Tools) and grown at 37 °C with shaking for 8 h to overnight. 1 ml of cells were pelleted and washed 3 times with LB and resuspended in 0.1 ml of LB. 2.5 µl of 10 µg/ml Click-iT Alexa Fluor 488/555 sDIBO Alkyne (for epifluorescence) or Alexa Fluor 647 sDIBO Alkyne (for 4Pi-SMS) (Thermo Fisher Scientific) were added to the resuspended cells, and the mixture shaken at 37 °C overnight. Cells were pelleted and washed 3 times with 1× PBS and resuspended in 50 µl of 1× PBS. 2 to 5 µl of the labeled cells were placed on 1% agar pad in PBS created in a chamber using Gene Frame (Thermo Fisher Scientific). 10 µl PBS was subsequently added to the chamber and a freshly cleaned coverslip placed on the agar pad to seal the chamber. For 3D-dSTORM experiments, 5 mM KDO-Azide was used instead of 1 mM. Subsequently, cells were washed and diluted to an OD_600_ of 0.05 and the KDO-Azide step was repeated. For experiments in which LPS distribution was tracked during cell growth, the duration of labeling with Alexa Fluor 488 sDIBO Alkyne was 2 h.

### Coverslip Preparation for PALM and 4Pi-SMS Microscopy.

#1.5 high precision coverslips were sonicated at 44 kHz, 50 °C for 15 min in a solution of 1 M KOH. Coverslips were then transferred into a pure MiliQ filtered water and sonicated at 44 kHz, 50 °C for 15 min, this was repeated three times. After the third sonication in MiliQ filtered water, coverslips were transferred to 70% ethanol for storage.

### Microscopy and Image Acquisition.

#### Widefield fluorescence microscopy.

All widefield fluorescence microscopy was conducted on an Oxford NanoImager-S with a 100× 1.49 NA oil immersion objective. For GFP and AF488 labeled proteins, excitation was conducted using a 473 nm laser line at 20 mW and light was collected between 500 to 550 nm. For mCherry labeled proteins, excitation was conducted using a 561 nm laser line and light was collected between 575 to 616.5 nm and 665.8 to 693.4 nm. The excitation laser power and exposure time used were dependent on the sample being imaged. For dual color fluorescence imaging, the order of imaging and acquisition setup was set to minimize bleaching or overlap.

#### OmpF PALM microscopy.

All PALM experiments were conducted on an Oxford NanoImager-S with a 100× 1.49 NA oil immersion objective, cells imaged in PALM were fixed with formaldehyde and imaged in PBS. PALM images were collected using two distinct methods that differed in the modulation of the 405 nm activation laser. In the first method, the 561 nm excitation laser was on constantly throughout the experiment at between 20 to 40 mW, the 405 nm activation laser was also kept on throughout the experiment at low levels (beginning at less than 0.1 mW) and was steadily increased over the course of the experiment to account for the photobleaching of fluorophores. The second method involved using constant illumination from the 561 nm excitation laser between 20 to 40 mW. The 405 nm activation laser was manually pulsed between 0.1 to 1 mW to sparsely activate fluorophores. Exposure times varied between 50 to 100 ms and a maximum of 20,000 frames were collected.

#### 4Pi-SMS imaging.

3D-STORM imaging of LPS was performed on stationary phase and exponentially growing cells. In both cases, LPS labeling with KDO-azide/Alexa Fluor 647 sDIBO Alkyne was done in stationary phase, as described above. For distributions in exponentially growing cells, labeled cells were exposed to a brief period of growth in LB media before imaging. Cells were pelleted and washed three times with 1× PBS. 10 µl of the washed labeled cells in 1× PBS were then placed on Cell-Tak coated coverslips for 5 to 10 min for dSTORM imaging. Cells were then gently spread using nitrogen flow and washed with PBS. Sparse fiducial markers, 100 nm crimson beads, were added to the sample coverslips before washing thoroughly. 100 µl dSTORM buffer was added to the top of sample attached coverslip before adding another clean dry coverslip to form a sandwich. Two-component silicone-based adhesive, TwinSil (Picodent, 1,300, 1,000) was used to seal the sandwich on an aluminum holding ring before transferred to the microscope. The buffer and cells were sealed between the two coverslips for dSTORM imaging of multiple fields of views. For imaging, 7.5 kW cm^−2^ laser power density was used to excite the sample in a 25 × 25 µm^2^ field of view area. Image data were acquired at 100 fps using a sCMOS camera, Hamamatsu ORCA-Flash4.0 V3 Digital CMOS (ORCA FLASH C11440-22CU, Hamamatsu Photonics). Typically, 30 to 60 k frames were acquired to reconstruct the final image using custom-written MATLAB-based analysis software. Details of the 4Pi-SMS superresolution imaging system have been described previously (39).

#### 3D-SIM imaging.

Cells were imaged using Deltavision OMX-SR microscopy system (GE Healthcare) equipped with four laser lines (405, 488, 568, and 640 nm), pco.edge 4.4 sCMOS cameras (PCO) and a 60× oil-immersion objective (Olympus PlanApo 1.42 NA). 1.512 index refraction immersion oil was used for AF488 labeled proteins. Images were acquired by imaging a 42 μm × 42 μm area with the 488 nm laser (2.7 mW, 200 ms exposure). Image stacks of 1 to 1.5 μm thickness were taken with 0.125 μm z-steps and 15 raw images (three angles and five phases per angle) per z-step and a 3D structured illumination with a stripe linewidth of 213 nm. The SIMcheck plugin (ImageJ) was used to assess data quality of SIM images. Image stacks were reconstructed using Deltavision softWoRx 7.2.0 software with a Wiener filter of 0.003 using wavelength-specific experimentally determined OTF functions.

#### FRAP experiments.

FRAP experiments were conducted using a Zeiss LSM 780 microscope. mCherry and AF488 labeled OmpF and LPS were visualized using a 100× 1.4 NA oil immersion objective lens. 2% 561 nm and 488 nm laser lines, respectively, were used for imaging. Transmitted light was collected to generate a DIC image of the cell. Bleaching was conducted by scanning over a 30 × 70 px region at the center or pole of the cell at 100% laser power (561 nm for mCherry, 488 nm for AF488) for 15 iterations. A single prebleach image was collected then bleaching conducted, after which an image was collected every 4 s for up to a total of 120 s. Transmitted light was collected to generate a DIC image of the cell for each frame. Analysis of FRAP data was conducted using FIJI. 2 channel image stacks were opened in FIJI. For each FRAP image sequence three 50 × 50 px areas were defined: target area, background area, and reference area. The target area was defined as the bleached area of the cell and the background taken from a random area of the image. The reference was defined as the pole of the cell that was not bleached and so could be used to correct for small amounts of Z-drift and whole cell bleaching throughout the course of the experiment.

### Microfluidics Setup and Experimental Procedures.

A Mother machine device was used to conduct microfluidics experiments, consisting of 100 × 25 μm (width × depth) media flow channels with perpendicular trenches of 1.2 × 1.2 × 25 μm (width × depth × length) in which bacteria were grown. As described in previous studies (51), microfluidic chips were made in Polydimethylsiloxane (PDMS) from a silicon wafer mold (ConScience AB), by mixing monomer and curing agent 1:10 (Dow Corning Sylgard 184 kit Farnell electronics, 1667370). After removing air bubbles under vacuum, the PDMS was cured on the mold at 65 °C for 2 h. Solidified PDMS was separated from the silicon wafer, cut into the correct size of one chip with a scalpel, and a 0.75 mm biopsy punch then used to create holes for media inlet and outlet. Next, the device was washed 2 to 3 times with 100% ethanol and dried with nitrogen gas in between washes. The device was bonded to a microscope coverslip (No 1.5 24 × 50 mm, VWR 631‐0147) that had been cleaned before by sonication in acetone for 20 min, followed by 2 washes in dH_2_O, followed by 20 min sonication in 100% isopropanol. The coverslips were dried using nitrogen gas. Bonding of the PDMS chip to a cleaned coverslip was done using air plasma treatment (Plasma Etch PE‐50) and heating at 95 °C for 30 min.

Microfluidics experiments were performed with a derivative strain from *E. coli* AB1157 that is nonmotile due to deletion of the *flhD* gene rendering cells unable to swim out of the trenches. Cells were grown and labeled as described above with the extra addition of 0.85Â mg/ml of surfactant pluronic F127 (Sigma-Aldrich, P2443‐250G) to avoid cell aggregation in the PDMS device. After LPS labeling, cells were loaded into the cover glass-bonded microfluidic chip by pipetting into the inlet hole and the device was then centrifuged (10 min × 2348 g) to push the cells into the growth channels. The device was placed on the microscope and Tygon Microbore silicon tubing (VWR ND 100‐80/0.508*1.524) used to link the device to a 50 mL syringe filled with M9G media containing 85 mg/mL pluronic F127. Medium was flowed into the device via a syringe pump (ALADDIN‐220, World Precision Instruments). The pumping rate was set at 2.5 mL/h for 10 min after loading the cells to flush out any cells in the media flow channel, then lowered to 0.5 mL/h for the remainder of the microscopy data acquisition.

Images were acquired using a Nikon Ti Eclipse inverted fluorescence microscope with a perfect focus system, oil immersion objective 100× NA1.4, motorized stage, sCMOS camera (Hamamatsu Flash 4), LED excitation source (Lumencore Spectra X), and incubation chamber maintained at 37 °C. Time‐lapse movies were taken with 10 min time intervals using the NIS‐Element software (Nikon) with an exposure time of 100 or 200 ms (λ = 471 nm) and a LED intensity set at 30% maximal output.

### Image and Data Analysis.

#### Production of fluorescence intensity heatmaps and kymographs.

Binary image generation was conducted in either FIJI or with custom-written Python Scripts using a simple user-defined threshold intensity. In all cases, binary image clean-up to remove erroneously segmented noise and cell clumps was conducted in Python. Noise and cell clumps were eliminated by interrogating 3 variables for each segmented object: solidity, eccentricity, and size. Average cell fluorescence intensity calculations were conducted using custom-written Python scripts. These scripts took as inputs fluorescence images and their cognate binary images. The pixel intensities making up each cell were measured by referencing the binary image in which all the cells had been identified. Plotting of the final data was conducted using the Python library matplotlib. For creating 2D- fluorescence intensity heatmaps, cell segmentation, bounding box identification, cropping, and normalization was performed with custom-written Python scripts. Average 2D fluorescence intensity distributions were generated through FIJI average Z projection. Upon generation of normalized cells for 2D fluorescence intensity heatmaps, the major axis length for each cell was recorded. Kymographs were generated by taking the average cell intensity profile along the length of the normalized cell and representing this profile as a single column of pixels, these were then sorted by major axis length from the shortest cell on the left to the longest cell on the right.

#### Two color fluorescence distribution plots.

Measurement of individual fluorescent intensity profiles and calculation of the normalized fluorescence distribution of OmpF and LPS were carried out individually. After aligning the two channels the fluorescence intensity profile and normalization of the long axis between 0 to 100 was automated using the MicrobeJ plugin (v5.13 m). After integration of profiles from all cells, the value at the poles was set to 1 and the remaining values were normalized accordingly. Normalization was done using Excel and the data were plotted in GraphPad Prism 8 software.

#### Integrating the localization of LptD clusters.

Integrating LptD distribution from multiple cells was carried out as described previously for BamA (21). Integrated localization maps of LptD clusters were created using the ImageJ plugin MicrobeJ (v5.13 m) (52). For the detection of LptD clusters with MicrobeJ, Unsharp Mask filter was applied (Radius 2 px, Mask weight 0.5). Cells and maxima points detection was then carried out using the MicrobeJ plugin; in some cases, the autosegmentation was manually corrected to exclude improperly detected or clustered cells.

#### Analysis of the LPS signal and cell extension in microfluidics experiments.

For the analysis of LPS signal and distribution, images from different time points were first compared to identify potential drift. When drift was detected images from all time points were aligned before further analysis. Subsequently, images were segmented and the average fluorescence intensity of each cell was calculated for every time point and the population average was normalized to the average at T_0_. Separate analysis was carried out for mother cells and for nongrowing cells. Mother cells were defined as the one cell which remains closest to the dead-end of the growth channel. Nongrowing (presumed dead) cells were defined as cells not showing significant extension throughout the experiment. For analyzing cell extension, the distance between the two poles of the cell of interest was manually measured at each time point using a merged transillumination-fluorescence image. To calculate the extension rate, the change in cell length was divided by the time difference (ΔL/ΔT).

### Statistical Analysis of 2D OmpF PALM Data and 3D Spatial Statistics of LPS Distribution.

#### Spatial statistics of OmpF distribution.

Before applying spatial statistics analysis, compounded localizations from OmpF PALM datasets were removed using a rudimentary single particle tracking program. A square window of dimensions 50 × 50 nm is scanned over the localizations in the order in which they were collected. If only one localization was present in the 50 × 50 nm region, the window was then moved to the next localization. If the window encountered a region in which multiple localizations were present, the frame number in which each localization within the window appeared was extracted. Frame numbers were checked to identify localizations that appeared in consecutive frames. These consecutive localizations were grouped together as representing a single OmpF protein. Consecutive localizations are then treated as single particle tracks and the centroid of these tracks taken as the position of the OmpF protein present. This process was repeated for all localizations in the dataset, the resultant XY coordinates therefore represent locations of OmpF proteins in the OM. Following removal of compounded localizations, spatial statistics analysis was carried out. Ripley’s K function provides a measure of the mean number of OmpF proteins within distance r of an arbitrary OmpF protein. From this can be derived the L function, which linearizes the K function, and the H function, which recalibrates it such that CSR patterns take a value of H around zero for all distances r. OmpF K, L, and H functions were compared to simulation envelopes for the most extreme values of these functions computed from a set of simulated CSR patterns. These computations were performed using the spatstat (2.1-0) package in R (4.1.0). Data were exported and plotted in Python (3.9.1 64-bit) matplotlib (3.3.3).

#### 3D spatial statistics analysis of LPS distribution.

Bacterial cells were modeled as a pill shape; a cylinder with a hemisphere cap at each end for both stationary phase and exponential phase cells (*SI Appendix*, 3D *LPS Spatial Statistics Supplementary file*). The pill that best fits the LPS coordinates was computed by minimizing the sum of the squared perpendicular distances from the localizations to the candidate. Estimates of the K function were computed directly on the pill shapes as per the methodology of (41). The K function applied here was a curved surface analogue of Ripley’s K function for planar surfaces. It provides a measure of the mean number of LPS molecules within a distance r of an arbitrary LPS, when mapped onto a sphere with unit radius. It is monotonically nondecreasing because the number of LPS molecules within a distance r of an arbitrary LPS will always stay constant or increase as r increases. From this, we also derive the P function. This linearizes and recalibrates the K function such that estimates of P are close to zero if LPS molecules are CSR (i.e., do not exhibit clustering). If the P function is significantly above zero then this provides strong evidence for clustering. The K and P functions estimated from the LPS coordinates were then compared to K and P functions computed from 999 simulated CSR (homogeneous Poisson) patterns on the same shape and with the same number of molecules. Fig. 1*C* shows the LPS coordinates have a K and P function that are significantly above the envelopes formed from the most extreme CSR K and P functions. To account for the possibility that some individual LPS molecules have two localization coordinates in the data, envelopes were also computed by simulating additional “double blinks” for elements of the CSR patterns (see *SI Appendix* for further details). Again, the LPS coordinates have a K and P function that are significantly above the envelopes, thus ruling out that the inferred clustering is due to (double blink) imaging artifacts. (41).

## Supplementary Material

Appendix 01 (PDF)

Movie S1. **Super-resolution microscopy reveals the surface distribution of OmpF**. Super-resolution reconstruction of OmpF organisation in *E. coli* MG1655 imaged by PALM. Shown are whole cell distribution followed by increased magnifications of specific OM regions highlighting OmpF clustering.

Movie S2. **Tracking LPS labelling during cell growth in the Mother Machine setup**. Continuous tracking of LPS distribution in microfluidic flow channels (Mother Machine) for growing *E. coli* cells pulse-labelled with KDO-azide and Alexa Fluor-488 sDIBO. Shown is a time-lapse movie of a single field of view containing 20 growth channels. Frames were taken at 10min intervals.

Movie S3. **Tracking general cell growth in the Mother Machine setup**. Continuous tracking of cell growth in microfluidic flow channels (Mother Machine) for *E. coli* cells pulse-labelled with KDO-azide and Alexa Fluor-488 sDIBO. Shown is a time-lapse movie of the epifluorescence channel allowing better comparative tracking of cell elongation and division. The presented field of view and imaging intervals are identical to Movie 2.

## Data Availability

Raw data are available in Zenodo repository (microscopy, microfluidics, LPS spatial data and associated statistical analysis) https://doi.org/10.5281/zenodo.14842881 (53). All other data are included in the article and/or supporting information. Materials are available from corresponding authors.
