# Supplementary material for: Immobile lipopolysaccharides and outer membrane proteins differentially segregate in growing Escherichia coli
Source: Proc Natl Acad Sci U S A. 2025 Mar 3;122(10):e2414725122. doi: 10.1073/pnas.2414725122 (PMC11912417; doi:10.1073/pnas.2414725122)
Supplement: Supplementary file 1 — Appendix 01 (PDF) [file pnas.2414725122.sapp.pdf]

Immobile lipopolysaccharides and outer membrane proteins differentially segregate in growing *Escherichia coli*

SI Appendix

<u>This document contains:</u>

3D LPS Spatial Statistics Supplementary File

Supplementary Figures 1-16

Supplementary Movies Legends 1-3

Supplementary Tables 1-4

Supplementary References

3D LPS Spatial Statistics Supplementary File

1 Preprocessing data

To approximate the surface of the bacteria we use a *pill*. A pill is defined as a shape with a cylindrical body that has hemi-spherical caps at each end and is commonly used to approximate the shape of different microbes. From the microscope we collect localisation of lipids in $\mathbb{R}^3$ and use this to fit the pill. The steps in our pipeline, as depicted in Supplementary Figure 4, are as follows:

1. The raw (x, y, z) localisation data is plotted.
2. Based on visual inspection, localisations that are too distant from where the surface is presumed to be are disregarded.
3. The pill that most closely matches the data is fit by a minimising the sum of the squared distances from the localisations to the shape. Initial values of the optimisation algorithm are based on visual inspection..
4. Localisations in the raw data are then mapped to their closest point on the surface of the fitted pill.
5. Finally to remove erroneous points that were mapped to an area outside of the observation window, using visual inspection, we set limits on the x-coordinates of the points.

1.1 Fitting the pill shape

Let the raw data be represented by $X = \{\mathbf{x}_1, \ldots, \mathbf{x}_n\}$, where n is the number of localisations. The pill has two parameters describing it: h, half the length of the cylindrical body and r the radius of the hemi-spherical cap (and circular cross section of the cylinder). We shall denote the pill by the symbol $\mathbb{D}_{h,r}$. The equation defining the pill is,

$$g(\mathbf{x}) = \begin{cases} x^2 + y^2 + (z - h)^2 - r^2, & \text{for } h < z \leq h + r \\ x^2 + y^2 - r^2, & \text{for } -h \leq z \leq h \\ x^2 + y^2 + (z + h)^2 - r^2, & \text{for } -(h + r) \leq z < -h, \end{cases}$$

where $\mathbf{x} = (x, y, z)^T$. In addition to this we allow the raw data to be rotated and translated in $\mathbb{R}^3$ leading to another 6 parameters that need to be fitted. Define $\boldsymbol{\mu} = (\mu_x, \mu_y, \mu_z)^T$ and

$\Gamma_{\theta,\phi,\psi}$ to be the translation vector and rotation matrix, respectively, where θ, ϕ, ψ define the angles used around each of the coordinate axes. These rigid motions are applied to X as $\Gamma(X - \boldsymbol{\mu}) = \{\Gamma(\mathbf{x} - \boldsymbol{\mu}) : \mathbf{x} \in X\}$, and the translated and rotated X is denoted $\tilde{X}(\boldsymbol{\mu}, \Gamma_{\theta,\phi,\psi}) \equiv \Gamma(X - \boldsymbol{\mu})$.

The shortest distance from points in $\tilde{X}(\boldsymbol{\mu}, \Gamma_{\theta,\phi,\psi})$ to the pill shape, defined by parameters h and r, take two possible forms. Let $\tilde{\mathbf{x}} \in \tilde{X}(\boldsymbol{\mu}, \Gamma_{\theta,\phi,\psi}) \equiv \Gamma(X - \boldsymbol{\mu})$ and suppose that its z-coordinate lies in $(h, h + r]$, i.e. in the top spherical cap. The shortest distance between $\tilde{\mathbf{x}}$ and the pill, denoted $\text{dist}(\mathbf{x}, \mathbb{D}_{h,r})$, is

$$\text{dist}(\tilde{\mathbf{x}}, \mathbb{D}_{h,r}) = \left|\sqrt{x^2 + y^2 + (z - h)^2} - r\right|, \quad \tilde{\mathbf{x}} = (x, y, z)^T.$$

Similarly we can obtain the distance for points $\tilde{\mathbf{x}} \in \tilde{X}(\boldsymbol{\mu}, \Gamma_{\theta,\phi,\psi}) \equiv \Gamma(X - \boldsymbol{\mu})$ that have z-coordinate in $[-(h + r), -h)$, i.e. the bottom spherical cap.

For points $\tilde{\mathbf{x}} \in \tilde{X}(\boldsymbol{\mu}, \Gamma_{\theta,\phi,\psi})$ that have z-coordinate in $[-h, h]$ the shortest distance is given by,

$$\text{dist}(\tilde{\mathbf{x}}, \mathbb{D}_{h,r}) = \left|\sqrt{x^2 + y^2} - r\right|, \quad \tilde{\mathbf{x}} = (x, y, z)^T.$$

Thus given the parameters $\boldsymbol{\theta} = (\mu_x, \mu_y, \mu_z, \theta, \phi, \psi, h, r)$ we can calculate the shortest distance between each point $\tilde{X}$ and the pill. We minimise the following criteria

$$\mathcal{L}(\boldsymbol{\theta}) = \sum_{i=1}^{n} \text{dist}^2\left(\Gamma_{\theta,\phi,\psi}\left(\mathbf{x}_i - (\mu_x, \mu_y, \mu_z)^T\right), \mathbb{D}_{h,r}\right)$$

to find the parameters $\boldsymbol{\theta}$. We use the general purpose optimiser `fminunc` available in `MATLAB` and set the initial values to $\boldsymbol{\theta}_{\text{init}} = (0, 0, 0, 0, 0, 0, h_{\text{init}}, r_{\text{init}})$ where h_{init} and r_{init} are chosen based on visual inspection.

2 Double blink model

Before discussing the mathematical details of the global and piecewise-analysis considered in the paper we first introduce the double blink model (DBM). Due to an artefact of the experimental instrument some fluorophores have the possibility of being localised twice and for any localisation there is some associated standard error. Based on the equipment, the probability of any lipid being localised twice is 0.134 whilst the standard error is between 10nm to 17nm depending on the axes. The DBM attempts to capture these two artefacts and account for them in the upcoming analysis to determine that the observed phenomena is due to some biological process as opposed to being a result of the multiple blinks and instrumentation.

2.1 Description

The DBM can be considered as a Neymann-Scott process [54]. We first have a parent process which follows some distribution over our pill, $\mathbb{D}$. In the upcoming sections this will typically be a model for complete spatial randomness (CSR): a model which states that positioning of the points are all independent of each other. Formally, the number of points across the pill follows a Poisson distribution and given this the points are uniformly distributed across its surface. Then for each parent it is localised either once with probability $p = 0.866$ and twice with probability $q = 1 - p = 0.134$. These localisations are then distributed normally around its parent point with standard error σ, i.e. with distribution

$$p(\mathbf{x}; \mathbf{k}) = \frac{1}{(2\pi\sigma^2)^{3/2}} \exp\left(\frac{-(\mathbf{x}-\mathbf{k})^T(\mathbf{x}-\mathbf{k})}{2\sigma^2}\right), \tag{1}$$

where $\mathbf{x}, \mathbf{k} \in \mathbb{R}^3$ and $\mathbf{k}$ is the parent point. The localisations are then mapped back to their closest point on the pill. Supplementary Figure 5 displays an example of the DBM and describe how it can be simulated.

2.2 Estimating intensity for global analysis

In the analysis presented in Section 3.1, it shall be necessary to simulate from the DBM with a CSR parent process, as specified by the null hypothesis being tested. Under this assumption we need to be able to control the expected number of points such that, on average, we obtain the same number as was observed in the data. Let K be the parent process with intensity ρ_K, let the corresponding DBM be X, and the window of observation be $W \subset \mathbb{D}$.

Let us consider the pattern as a marked point process such that the offspring points $M_{\mathbf{k}} \equiv \{Y_{i,\mathbf{k}}\}_{i=1}^{N_k}$ for each $\mathbf{k} \in K$ are the marks associated to $\mathbf{k}$, where $N_{\mathbf{k}}$ is the number of offspring points associated to $\mathbf{k} \in K$ and $Y_{i,\mathbf{k}}$ is one of the offspring points with probability density function given by equation (1). Then we have that the expectation of $N_X(W)$, the

number of points in X that reside in W, is given as

$$\mathbb{E}[N_X(W)] = \mathbb{E}\sum_{\mathbf{k}\in K}\sum_{i=1}^{N_{\mathbf{k}}} \mathbb{1}[Y_{i,\mathbf{k}} \in W'].$$

Then by the Campbell-Mecke Theorem for marked point processes,

$$\begin{aligned}
\mathbb{E}[N_X(W)] &= \int_{\mathbb{D}} \mathbb{E}\left[\sum_{i=1}^{N_{\mathbf{k}}} \mathbb{1}[Y_{i,\mathbf{k}} \in W']\right] \rho_K d\mathbf{k} \\
&= \int_{\mathbb{D}} \mathbb{E}_{N_{\mathbf{k}}}\left[\mathbb{E}\left(\sum_{i=1}^{N_{\mathbf{k}}} \mathbb{1}[Y_{i,\mathbf{k}} \in W'] \middle| N_{\mathbf{k}}\right)\right] \rho_K d\mathbf{k} \\
&= \int_{\mathbb{D}} \mathbb{E}_{N_{\mathbf{k}}}\left[\sum_{i=1}^{N_{\mathbf{k}}} \mathbb{E}\left(\mathbb{1}[Y_{i,\mathbf{k}} \in W'] \middle| N_{\mathbf{k}}\right)\right] \rho_K d\mathbf{k} \\
&= \int_{\mathbb{D}} \mathbb{E}_{N_{\mathbf{k}}}\left[\sum_{i=1}^{N_{\mathbf{k}}} \mathbb{P}\left(Y_{i,\mathbf{k}} \in W'\right)\right] \rho_K d\mathbf{k}.
\end{aligned}$$

We can now drop the subscript i since the probability measure is the same for each offspring, giving

$$\begin{aligned}
\mathbb{E}[N_X(W)] &= \int_{\mathbb{D}} \mathbb{E}_{N_{\mathbf{k}}}\left[\sum_{i=1}^{N_{\mathbf{k}}} \mathbb{P}\left(Y_{\mathbf{k}} \in W'\right)\right] \rho_K d\mathbf{k} \\
&= \int_{\mathbb{D}} \mathbb{E}_{N_{\mathbf{k}}}\left[\sum_{i=1}^{N_{\mathbf{k}}} 1\right] \mathbb{P}\left(Y_{\mathbf{k}} \in W'\right) \rho_K d\mathbf{k} \\
&= \int_{\mathbb{D}} \mathbb{E}_{N_{\mathbf{k}}}\left[N_{\mathbf{k}}\right] \mathbb{P}\left(Y_{\mathbf{k}} \in W'\right) \rho_K d\mathbf{k}.
\end{aligned}$$

Since $\mathbb{E}_{N_{\mathbf{k}}}[N_{\mathbf{k}}] = p + 2(1-p) = 2-p$,

$$\mathbb{E}[N_X(W)] = (2-p)\rho_K \int_{\mathbb{D}} \mathbb{P}\left(Y_{\mathbf{k}} \in W'\right) d\mathbf{k},$$

where p is the probability of each parent producing one localisation, $W' \subset \mathbb{R}^3$ is the volume such that, for every point $\mathbf{x} \in W'$, its closest point on $\mathbb{D}$ lies in $W \subseteq \mathbb{D}$, and $Y_{\mathbf{k}}$ are random variables with probability density function given by (1).

The final integral is not known analytically but can be approximated. Given the dimensions of the fitted pill typically far exceed the standard deviation of the localisation errors,

then $P(Y_{\mathbf{k}} \in W') \approx \mathbb{1}[\mathbf{k} \in W]$. In other words, those parent points that are observed in W produce offspring that are also in W. This is typical for most of the points except those near the edge of the window. This is also identical to the limit when σ goes to 0 and would be regarded the most clustered DBM. Thus we can approximate the expectation of the DBM as,

$$\begin{aligned}\mathbb{E}[N_X(W)] &\approx (2-p)\int_{\mathbb{D}} \mathbb{1}[\mathbf{k} \in W]\rho_K d\mathbf{k} \\ &= (2-p)\rho_K |W|,\end{aligned}$$

where $|W|$ is the area of W. Therefore in our simulations, when conducting the global analysis, we estimate the constant intensity, ρ_K, of the parent process as,

$$\hat{\rho}_K = \frac{N_X(W)}{(2-p)|W|}. \tag{2}$$

2.3 Estimating intensity for piecewise-analysis

For the piecewise-analysis presented in 3.2, we study the cylindrical and spherical portions of the pill separately. Again, it shall be necessary to simulate from the DBM with a CSR parent process, as specified by the null hypothesis being tested. Under this assumption we need to be able to control the expected number of points on each piece such that, on average, we obtain the same number as was observed in the data. To do so, we need to estimate of the constant intensity ρ_K of the parent process K, for each piece separately.

Let us consider the front cylinder, that is the points $\mathbf{x} \in X$ such that $x \geq 0$ and $-h \leq z \leq h$, where $\mathbf{x} = (x, y, z)^T$. We divide the pill into three parts: the cylinder, $\mathbb{D}_{\text{cyl}}$, the top cap, $\mathbb{D}_{\text{tcap}}$, and the bottom cap, $\mathbb{D}_{\text{bcap}}$. Let X again be our DBM and K the parent points then the expected number of points on $\mathbb{D}_{\text{cyl}}$ is,

$$\begin{aligned}\mathbb{E}[N_X(\mathbb{D}_{\text{cyl}})] &= \mathbb{E}\sum_{\mathbf{k}\in K}\sum_{i=1}^{N_{\mathbf{k}}} \mathbb{1}[Y_{i,\mathbf{x}} \in \mathbb{D}'_{\text{cyl}}] \\ &= (2-p)\int_{\mathbb{D}} P(Y_{\mathbf{k}} \in \mathbb{D}'_{\text{cyl}})\rho_K d\mathbf{k},\end{aligned}$$

where $\mathbb{D}'_{\text{cyl}} \subset \mathbb{R}^3$ is the volume such that for every point $\mathbf{x} \in \mathbb{D}'_{\text{cyl}}$ its closest point on $\mathbb{D}$ lies in $\mathbb{D}_{\text{cyl}}$. This result follows similar arguments to those made in the previous section. Note that the event $Y_{\mathbf{k}} \in \mathbb{D}'_{\text{cyl}}$ is identical to the event $Z_{\mathbf{k}} \in [-h, h]$ where $Z_{\mathbf{k}}$ is the z-coordinate of $Y_{\mathbf{k}}$. We can therefore replace $P(Y_{\mathbf{k}} \in \mathbb{D}'_{\text{cyl}})$ with $P(-h \leq Z_{\mathbf{k}} \leq h)$, and since P is a trivariate normal distribution with diagonal covariance matrix then marginally $Z_{\mathbf{k}}$ is univariate normal with standard deviation σ and mean given by the z-coordinate of $\mathbf{k}$, its parent point. Thus we can rewrite $\mathbb{E}[N_X(\mathbb{D}_{\text{cyl}})]$ as,

$$\mathbb{E}[N_X(\mathbb{D}_{\text{cyl}})] = (2-p)\rho_K \int_{\mathbb{D}} \Phi\left(\frac{h - \mathbf{k}_z}{\sigma}\right) - \Phi\left(\frac{-h - \mathbf{k}_z}{\sigma}\right) d\mathbf{k},$$

where $\mathbf{k}_z$ is the z-coordinate of $\mathbf{k}$ and Φ is the cumulative distribution function for a standard normal random variable. Notice though that we do not observe the process on all of the cylinder but instead a vertical segment, say $\mathbb{D}_{\text{cyl,vert}}$. By symmetry of the pill, for any vertical segment of the cylinder, the number of points on that segment must be proportional to the number of points across all of the cylinder. Furthermore, the constant of proportionality is $|\mathbb{D}_{\text{cyl,vert}}|/|\mathbb{D}_{\text{cyl}}|$, i.e. the proportion of the total area of the cylinder contained in the vertical segment. The expectation over the front vertical cylinder portion is therefore

$$\begin{aligned}\mathbb{E}[N_X(\mathbb{D}_{\text{cyl,vert}})] &= \frac{|\mathbb{D}_{\text{cyl,vert}}|}{|\mathbb{D}_{\text{cyl}}|}\mathbb{E}[N_X(\mathbb{D}_{\text{cyl}})] \\ &= \frac{|\mathbb{D}_{\text{cyl,vert}}|}{|\mathbb{D}_{\text{cyl}}|}(2-p)\rho_K \int_{\mathbb{D}} \Phi\left(\frac{h-\mathbf{k}_z}{\sigma}\right) - \Phi\left(\frac{-h-\mathbf{k}_z}{\sigma}\right) d\mathbf{k}.\end{aligned}$$

Since we observe $N_X(\mathbb{D}_{\text{cyl,vert}})$ we can estimate ρ_K as

$$\hat{\rho}_K = \frac{|\mathbb{D}_{\text{cyl}}|N_X(\mathbb{D}_{\text{cyl,vert}})}{|\mathbb{D}_{\text{cyl,vert}}|(2-p)\int_{\mathbb{D}} \Phi\left(\frac{h-\mathbf{k}_z}{\sigma}\right) - \Phi\left(\frac{-h-\mathbf{k}_z}{\sigma}\right) d\mathbf{k}}, \tag{3}$$

where the integral can be approximated using Monte Carlo integration with a uniform density over $\mathbb{D}$.

Similar arguments can be made for the estimation of the intensity of the parent process on the caps. The only caveat is that the symmetry arguments made for the cylindrical portion cannot be made for the spherical cap due to the shape of the observation window. Even though this does not hold we still apply a constant of proportionality given by the ratio of the area of the observation window on the cap over the total area of the cap. Simulations show that approximately on average we observe the same number of points in the simulates as the observed data. This leads to estimating the intensity on the caps as

$$\hat{\rho}_K = \frac{|\mathbb{D}_{\text{cap}}|N_X(\mathbb{D}_{\text{cap,window}})}{|\mathbb{D}_{\text{cap,window}}|(2-p)\int_{\mathbb{D}} \frac{1}{2}(1-[\Phi\left(\frac{h-\mathbf{k}_z}{\sigma}\right) - \Phi\left(\frac{-h-\mathbf{k}_z}{\sigma}\right)])d\mathbf{k}}, \tag{4}$$

where $\mathbb{D}_{\text{cap,window}}$ corresponds to the observation window on either the top or bottom cap of the pill. Again, the integral can be approximated using Monte Carlo integration with a uniform density over $\mathbb{D}$.

3 Spatial analysis

3.1 Global analysis

Typically exploratory data analysis for spatial point patterns is conducted using functional summary statistics such as Ripley's K-function [55, 54], but due to the irregularity of the pill these approaches, which frequently impose that the process is observed in $\mathbb{R}^d$, are not appropriate. Instead we shall follow the approach of [56] who, by mapping point processes onto the unit sphere $\mathbb{S}^2$, are able to test whether an observed point pattern has arisen from a CSR process. For theoretical details the reader is directed to [56].

If we assume that the process on the fitted pill $\mathbb{D}$ is CSR with constant intensity ρ, we can map the process to a Poisson process on the sphere by the function $f(\mathbf{x}) = \mathbf{x}/||\mathbf{x}||$ for $\mathbf{x} \in \mathbb{D}$ where $||\mathbf{x}||$ is the Euclidean distance of the point $\mathbf{x}$ to the origin. On the sphere, the Poisson process has inhomogeneous intensity function

$$\rho^*(\mathbf{x}) = \rho \det(J_f(\mathbf{x})), \tag{5}$$

where $J_f(\mathbf{x})$ is the Jacobian matrix of the function f and $\det(\cdot)$ is the determinant operator. We can therefore construct estimates of the spheroidal inhomogeneous K-function [57, 58] as,

$$\hat{K}_{\text{inhom}}(r) = \frac{1}{|f(W)_{\ominus r}|} \sum_{\mathbf{x} \in f(X) \cap (f(W)_{\ominus r})} \sum_{\mathbf{y} \in f(X) \setminus \{\mathbf{x}\}} \frac{\mathbb{1}[d(\mathbf{x}, \mathbf{y}) \leq r]}{\rho^*(\mathbf{x})\rho^*(\mathbf{y})},$$

where $\rho^*(\cdot)$ is given by (5), W is our observation window on $\mathbb{D}$, $f(W)$ is the mapping of the observation window onto the sphere, $f(W)_{\ominus r}$ is the erosion of $f(W)$ by r on the sphere [54], and $d(\mathbf{x}, \mathbf{y})$ is the shortest distance (the great circle length) between two points $\mathbf{x}, \mathbf{y} \in \mathbb{S}^2$. Since ρ in (5) is unknown we estimate it as $N_X(W)/|W|$. Supplementary Figure 6 displays an example of mapping the points from the pill to the sphere and highlights the irregularity of the new observation window $f(W)$ on the sphere. Thus we use Monte Carlo integration over the sphere with a uniform density to estimate $|f(W)|$.

In order to construct simulation envelopes [54] under the null hypothesis of CSR, we simulate a CSR process over $\mathbb{D}$ with constant intensity function $\hat{\rho} = N_X(W)/|W|$. These are then mapped to the sphere and their corresponding estimates of $K_{\text{inhom}}(\cdot)$ are constructed. Additionally, we also simulate from the DBM with the intensity of the parent process given by (2). These are also mapped to the sphere and their estimates of the inhomogeneous K-functions computed. By comparing the estimates of the inhomogeneous K-function for the observed data against those of the CSR and DBM simulates, we can test for whether the process exhibits homogeneity. We also examine the inhomogeneous P-function which can be estimated as

$$\hat{P}_{\text{inhom}}(r) = \left(\hat{K}_{\text{inhom}}(r)\right)^{1/2} - (2\pi(1 - \cos(r)))^{1/2}$$

and is a simple transformation of the inhomogeneous K-function [59, 56].

3.2 Piecewise-analysis

To conduct piecewise analysis, we divided the point pattern into four parts: the top and bottom spherical caps, and then the two cylindrical segments. By dividing the pill into these four constituent parts we can make use of traditional planar spatial analysis techniques for the cylindrical sections [55, 54], and existing spherical techniques for the caps [60]. The major benefit of the piecewise analysis is that distances are now on the same scale as the observed data whereas for the global analysis we mapped to the sphere.

On the cylindrical portions, we first map the points onto the plane using the transformation, $g : \mathbb{R}^3 \mapsto \mathbb{R}^2$,

$$g(\mathbf{x}) = \begin{pmatrix} r \sin^{-1}(x/r) \\ z \end{pmatrix}, \quad \mathbf{x} = (x, y, z)^T,$$

where r is the fitted radius of the pill. We then construct the L-function [54]. For the caps we consider the P-function [59, 60].

To construct the simulation envelopes under the null hypothesis of CSR, we simulate CSR processes with an intensity estimated specifically to the part under investigation. More explicitly, if we are looking at the front cylindrical portion, labelled $\mathbb{D}_{\text{cyl,front}}$, then we estimate the constant intensity function ρ by $\hat{\rho} = N_X(\mathbb{D}_{\text{cyl,front}})/|\mathbb{D}_{\text{cyl,front}}|$. We similarly simulate the DBM with constant parent intensity given by either (3) or (4) if the part under investigation is the cylinder or spherical cap, respectively.

To compliment this we also provide histograms of the nearest neighbour distances. We split the pattern into whether it lies on the cylindrical or spherical part of the pill and plot separate histograms for the two. To adjust for edge effects we discard points whose nearest neighbour is further then its shortest distance to the window edge. We compare these histograms to kernel density estimates of the nearest neighbour distribution for single simulates from either a CSR or DBM process. The kernel estimate uses a Gaussian kernel with the bandwidth selected assuming the data is normal [61]. To simulate the CSR process we use the estimate $\hat{\rho} = N_X(W)/|W|$ whilst for the DBM process we use (2) to simulate the parent process and then divide each into either cylindrical or spherical portions.

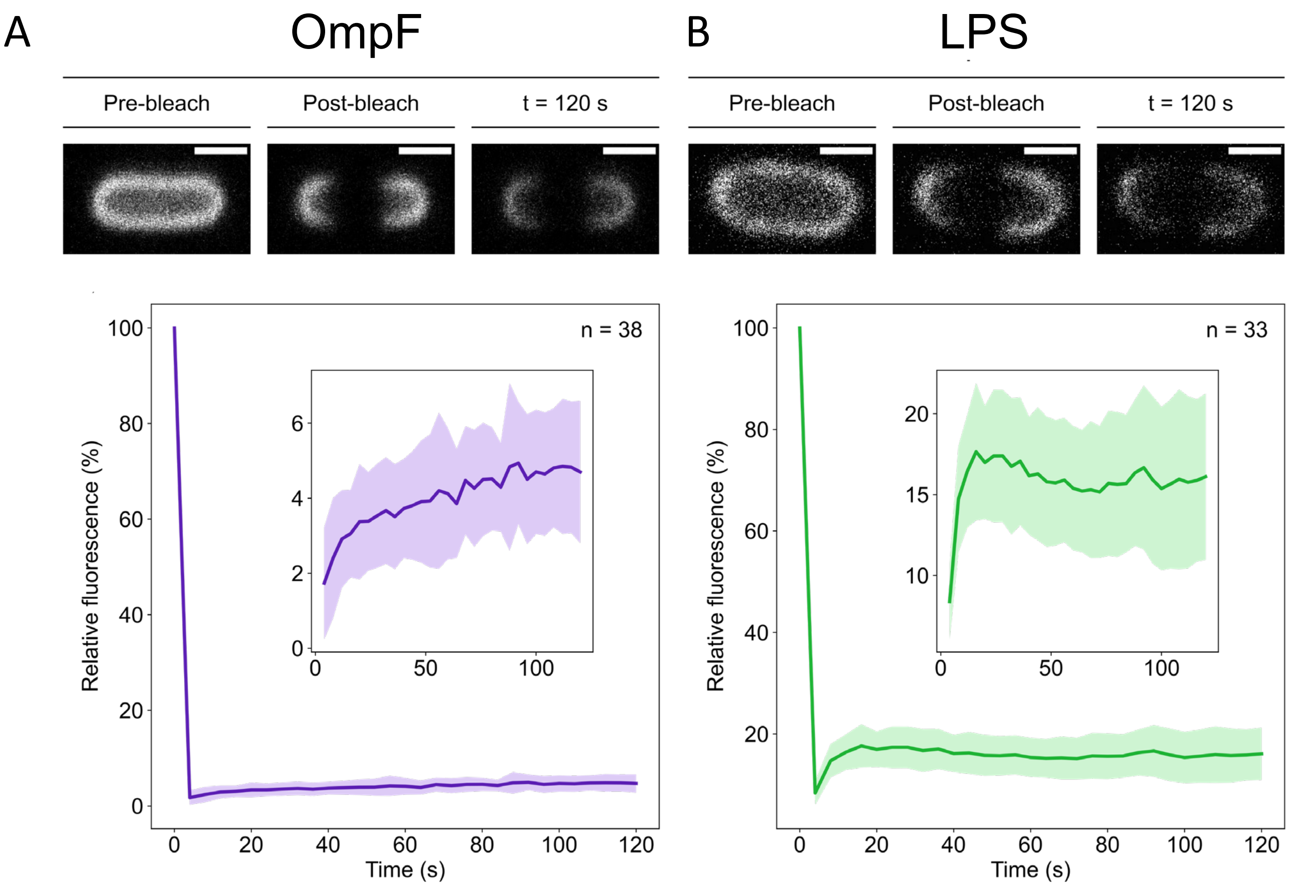

Supplementary Figure 1. Outer membrane OmpF and LPS are both immobile in live bacteria. Fluorescence Recovery After Photobleaching (FRAP) experiments carried using *E. coli* MG1655 cells post OmpF or LPS labelling (see Materials and Methods). **(A)** *Top panels*, Representative images of the OmpF FRAP experiment lasting 120 seconds. Cells were labelled with Colicin $N^{1\text{-}185}$ mCherry, the midcell region bleached and fluorescence recovery subsequently tracked. *Bottom panels*, Plot displaying the lack of fluorescence recovery. *Solid line*, average fit to the data; *shaded region*, (±) standard deviation. *Inset* shows blow-up of fit to the data over the time course. **(B)** *Top panels*, Representative images of the LPS FRAP experiment lasting 120 seconds. Cells were labelled with KDO-azide and Alexa Fluor-488 sDIBO, the midcell region bleached and fluorescence recovery subsequently tracked. *Bottom panels*, Plot displaying the lack of LPS fluorescence recovery. *Solid line*, average fit to the data; *shaded region*, (±) standard deviation. *Inset* shows blow-up of fit to the data over the time course. Scale bars 1µm.

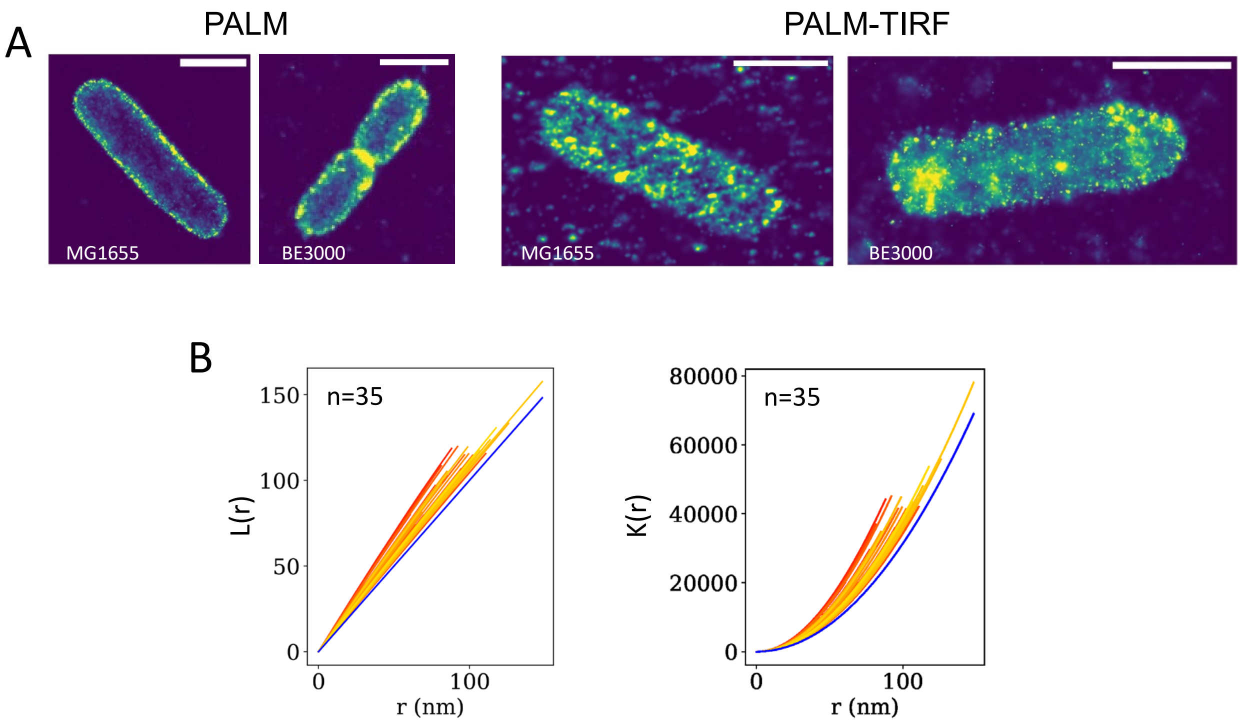

Supplementary Figure 2. Super-resolution fluorescence microscopy reveals the inhomogeneous surface distribution of OmpF.

Super-resolution reconstructions generated from datasets of 30,000 frames. *Left hand panels*, Representative images of OmpF organisation in *E. coli* MG1655 and BE3000 cells imaged by epifluorescence PALM. *Right hand panels,* Representative images of OmpF organisation in *E. coli* MG1655 and BE3000 cells imaged by PALM-TIRF. **(B)** Ripley's K (*right*) & L (*left*) functions were applied to processed experimental data from PALM datasets (*orange traces*). The expected function for 2D complete spatial random (CSR) coordinates was plotted (*solid blue line*). Simulations of 9,999 2D CSR datasets defined the upper and lower bounds of the CSR envelope (*light blue*)

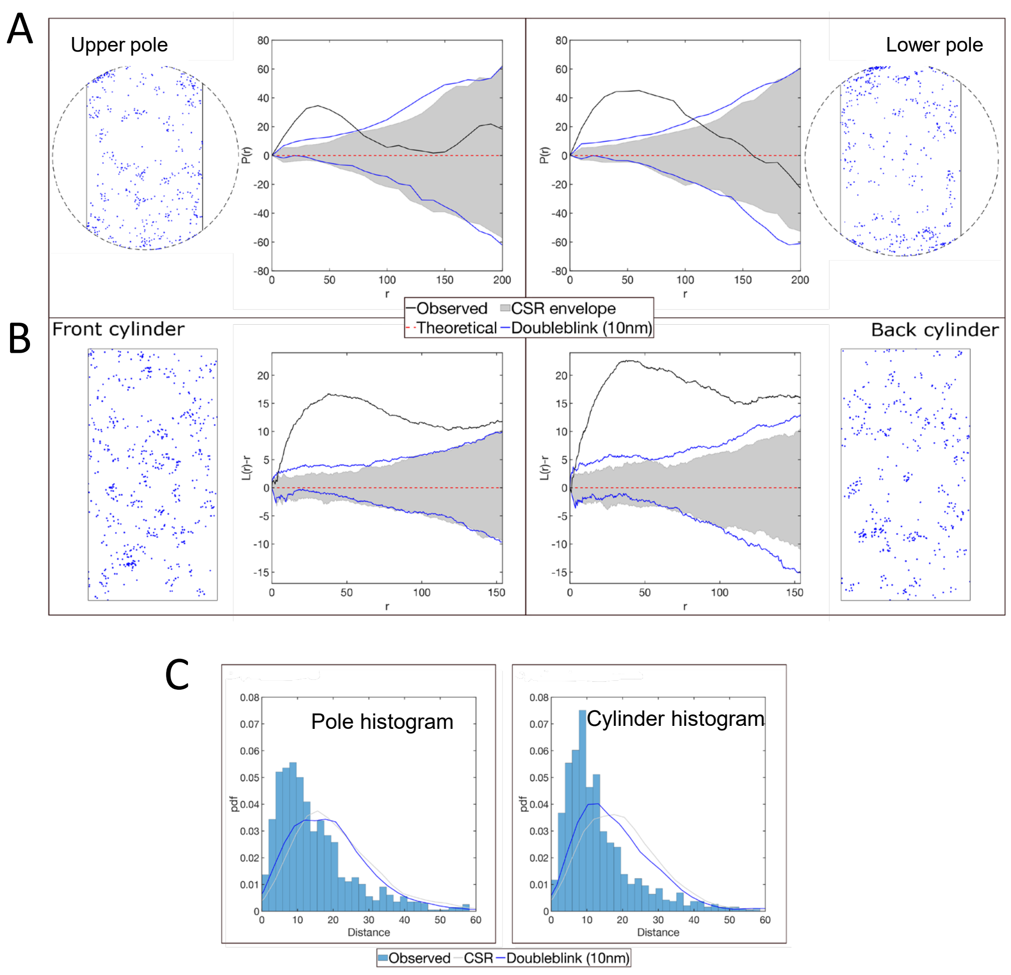

Supplementary Figure 3. LPS clustering at different regions of the bacterial cell surface.

Piecewise analysis of the localisations of the same cell as in Figure 1 which was modelled as a pill shape with cylindrical body and hemi-spherical upper and lower poles. **(A)** *Left hand panels*, Observed inhomogeneous P-function on the upper cell pole accompanying a plot of the LPS point pattern. *Right hand panels*, Observed inhomogeneous P-function on the bottom pole accompanying a plot of the LPS point pattern. **(B)** *Left hand panel*s, Observed inhomogeneous L(r) - r on the front face of the cylinder accompanying a plot of the LPS point pattern. *Right hand panels*, Observed inhomogeneous L(r) - r on the back face of the cylinder accompanying a plot of the LPS point pattern. Observed functional summary statistics are shown in *black*, theoretical values in dashed *red line,* simulation envelopes for the CSR in *grey*, and simulation envelope for the double blink model shown as *blue* lines. **(C)** Nearest neighbour histograms for the cell shown in Figure 1. *Left hand panel*, Nearest neighbour histogram for points on polar regions. *Right hand panel*, Nearest neighbour histogram for points on the cell cylinder. *Grey line*, the kernel density estimate for the nearest neighbour distribution of a CSR process. *Blue line*, kernel density estimate for the nearest neighbour distribution of the double blink model. See 3D LPS Spatial Statistics Supplementary for further details.

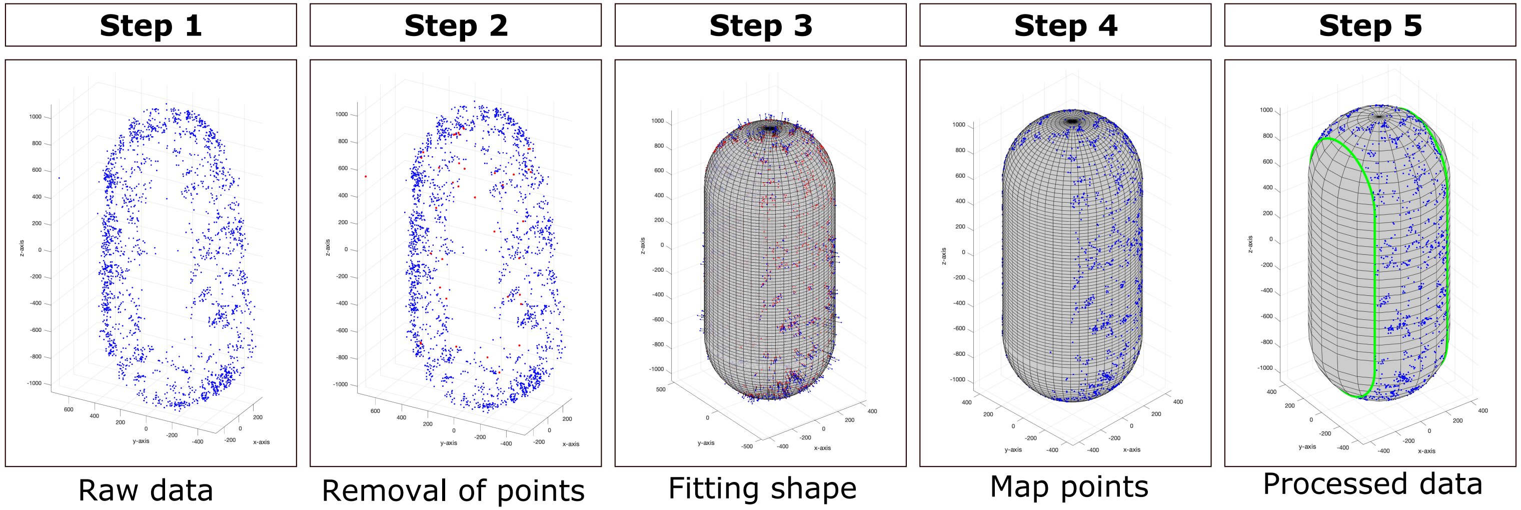

Supplementary Figure 4: Preprocessing steps for data: *step 1* - raw data obtained from the microscope (blue points), *step 2* - removing points that are considered too distant from the surface of the bacteria (points to be removed are indicated in red), *step 3* - using least squares to fit the pill shape parameters (raw data is indicated in blue, closest point on surface to each localisation is indicated in red), *step 4* - raw data point localisations are mapped to their closest point on the surface of the fitted pill, *step 5* - limits are set on the x-coordinate to remove points observed outside the observation window (limits are given by the green lines).

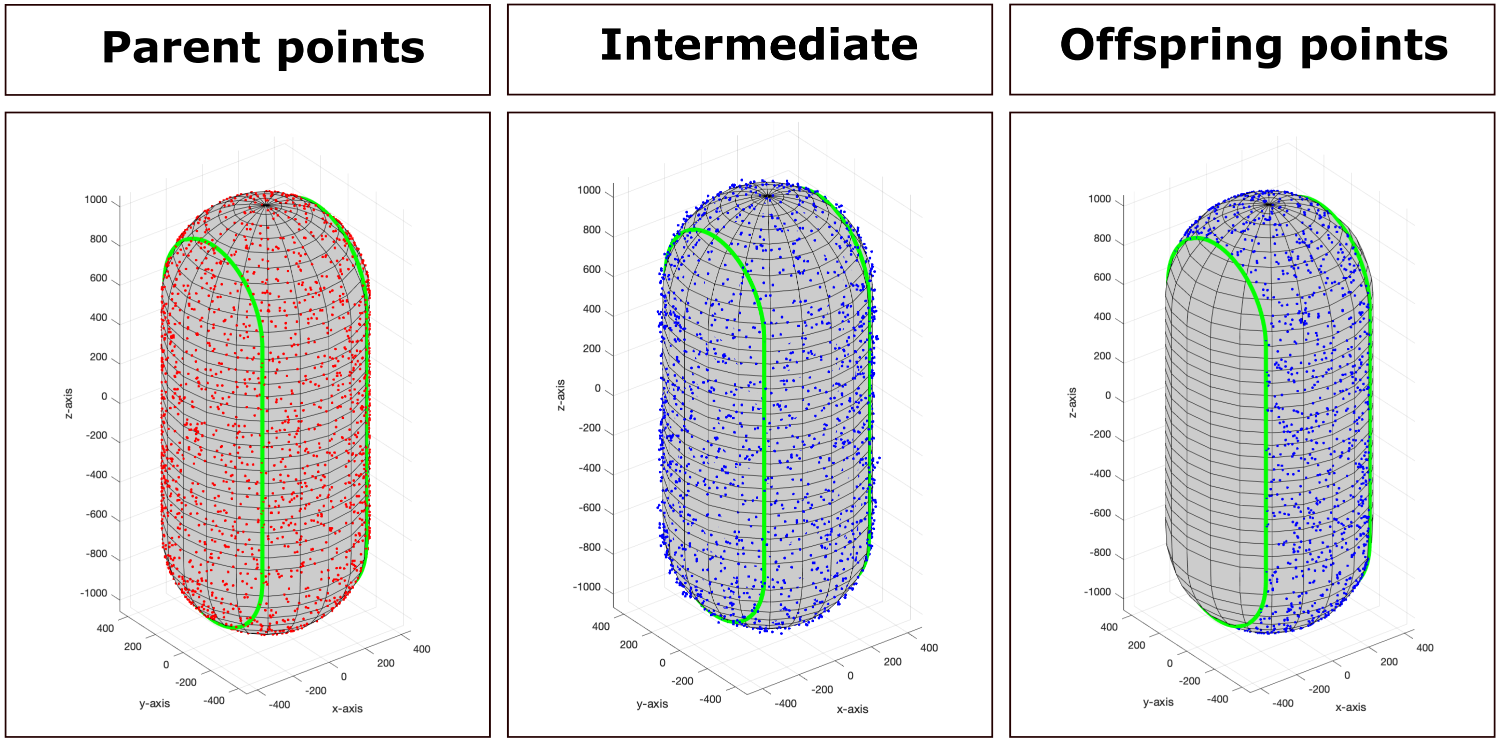

Supplementary Figure 5: Example of double blink model: *left figure* - provides a simulation of the parent points highlighted in red, *middle figure* - for each parent point either one or two offspring points are generated using the density given by Equation 1, *right figure* - the offspring points are mapped to their closest point on the surface of the pill and those points that fall outside the window of observation are discarded. The green lines show the observation window of interest on the pill.

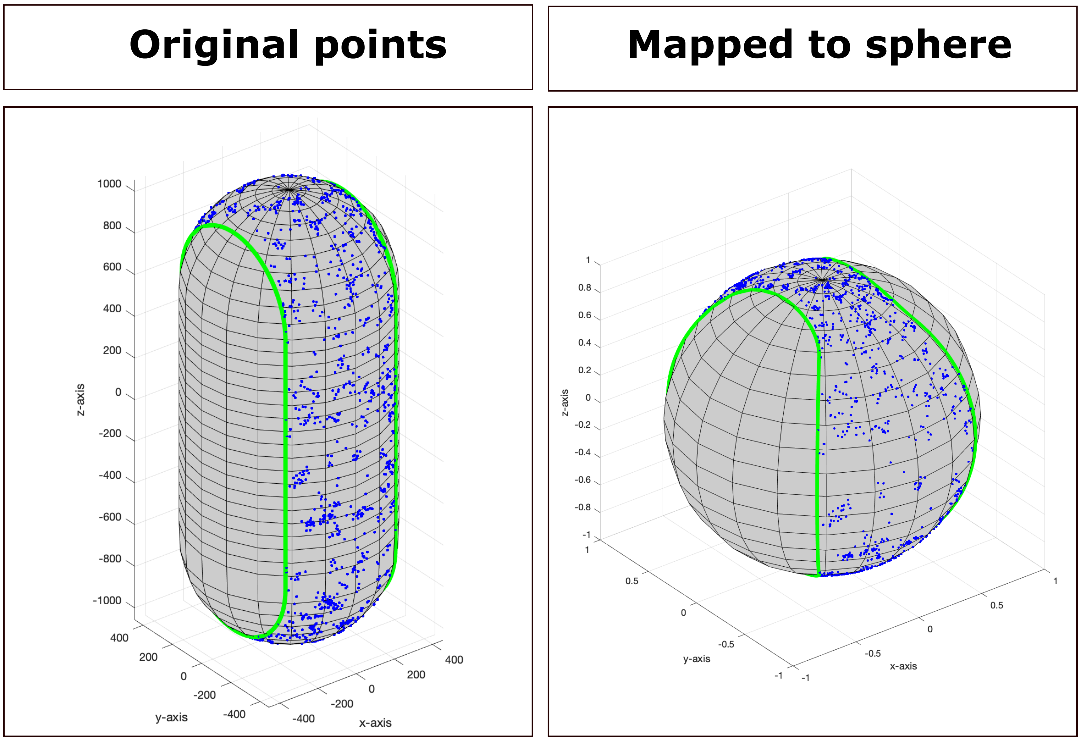

Supplementary Figure 6: Example of mapping point pattern to sphere: *left figure* - displays the original data on the fitted pill with the observation window indicated by the green line, *right figure* - shows the mapped point process onto the sphere with the corresponding window mapped and indicated by green on the sphere.

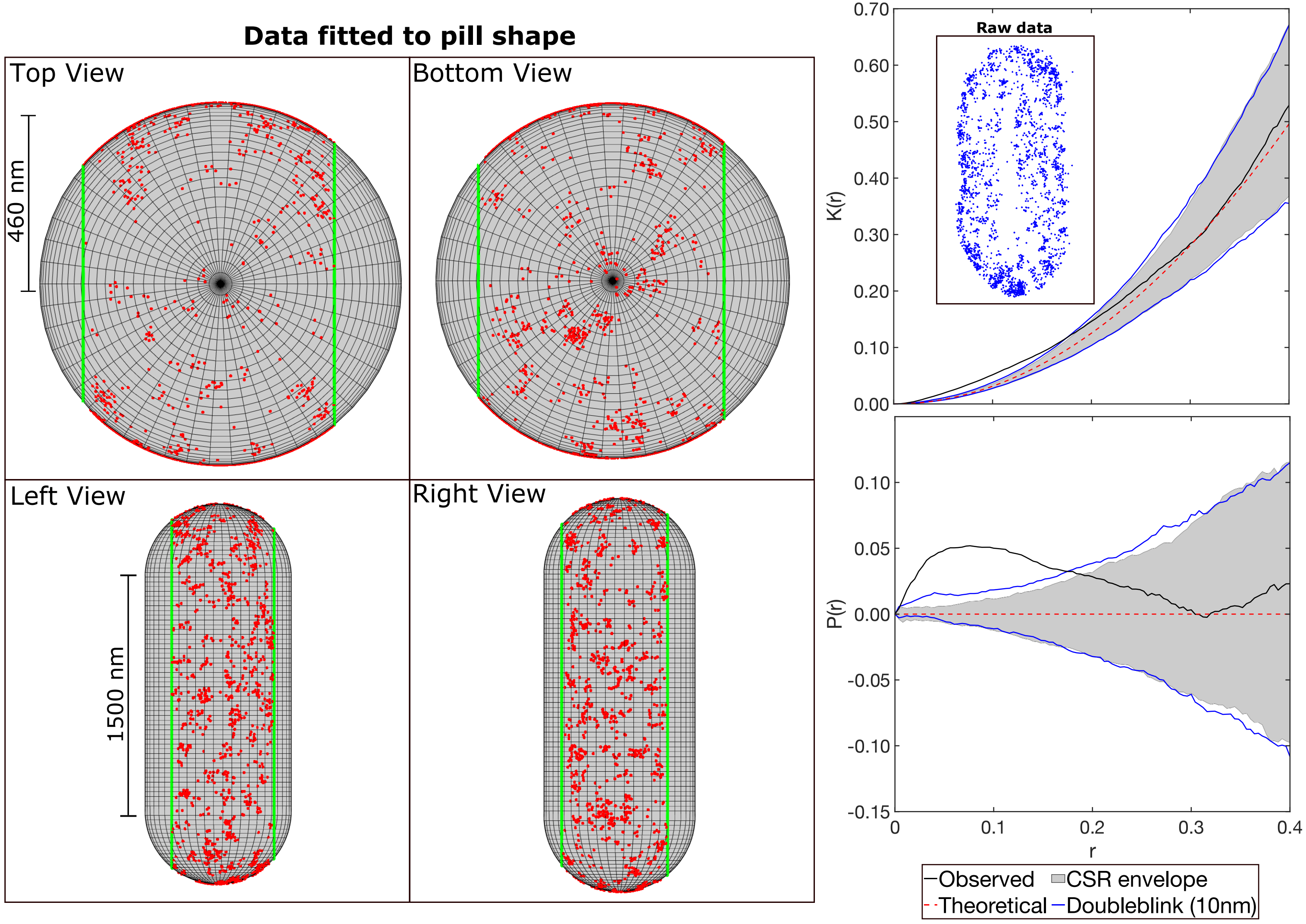

Supplementary Figure 7: **(Stationary)** Example global analysis: *left* - displays the points mapped to the fitted pill with dimensions of the pill provided, *top right* - plot of the observed inhomogeneous K-function (black line), with the theoretical value (dashed red line) and simulation envelopes for the CSR process (grey) and the DBM (blue lines), an insert of the raw data is also given, *bottom right* - plot of the observed inhomogeneous P-function (black line), with the theoretical value (dashed red line) and simulation envelopes for the CSR process (grey) and the DBM (blue lines)

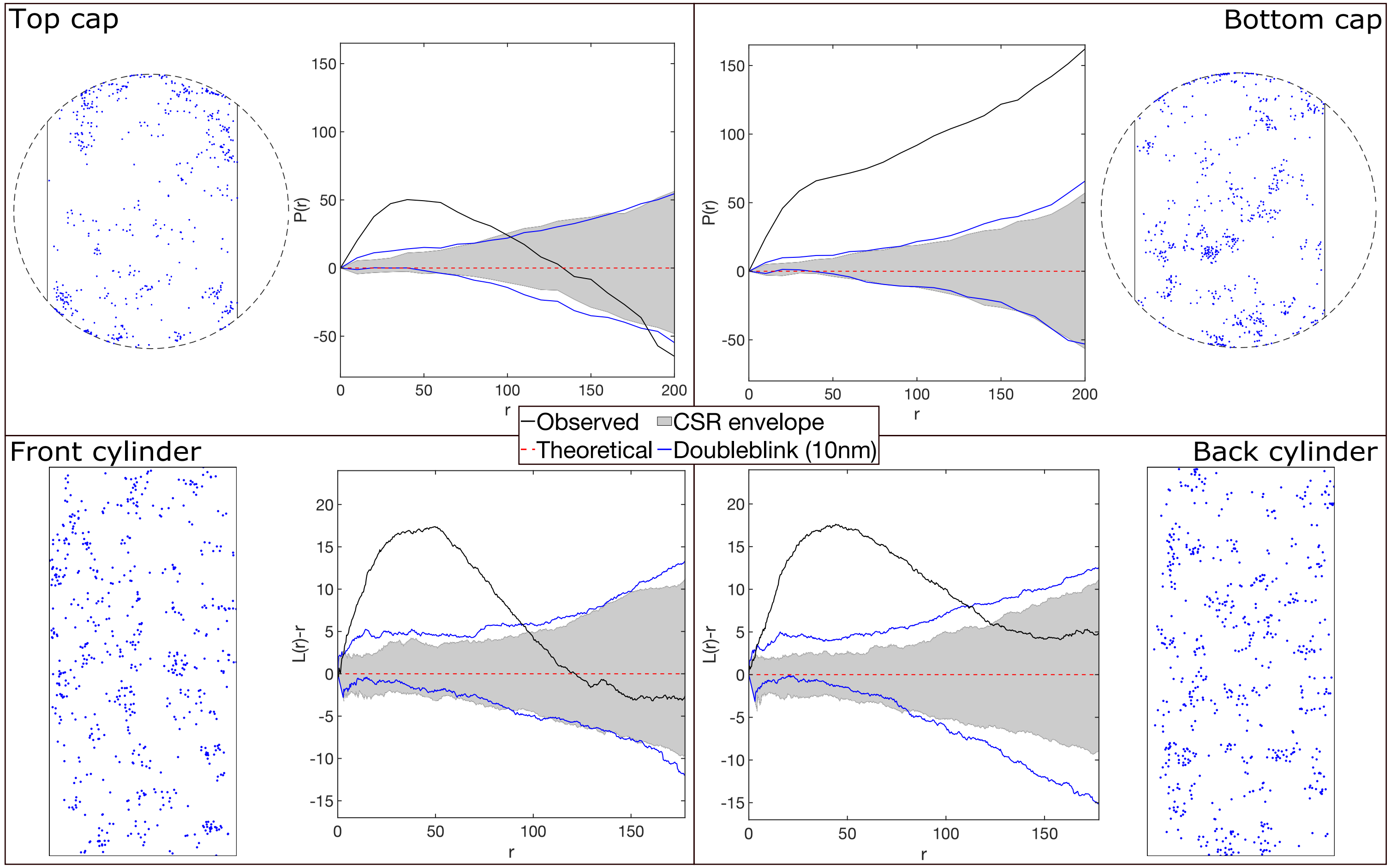

Supplementary Figure 8: **(Stationary)** Example piecewise analysis: *top left* - the observed homogeneous P-function on the top cap with a plot of the point pattern on the top cap, *top right* - the observed homogeneous P-function on the bottom cap with a plot of the point pattern on the bottom cap, *bottom left* - the observed homogeneous L-function on the front cylinder a plot of the point pattern on the front cylinder, *bottom right* - the observed homogeneous L-function on the back cylinder a plot of the point pattern on the back cylinder. Observed functional summary statistics is the black line, the theoretical value is the dashed red line and simulation envelope for the CSR process is grey and the simulation envelope for the DBM is given by the blue lines.

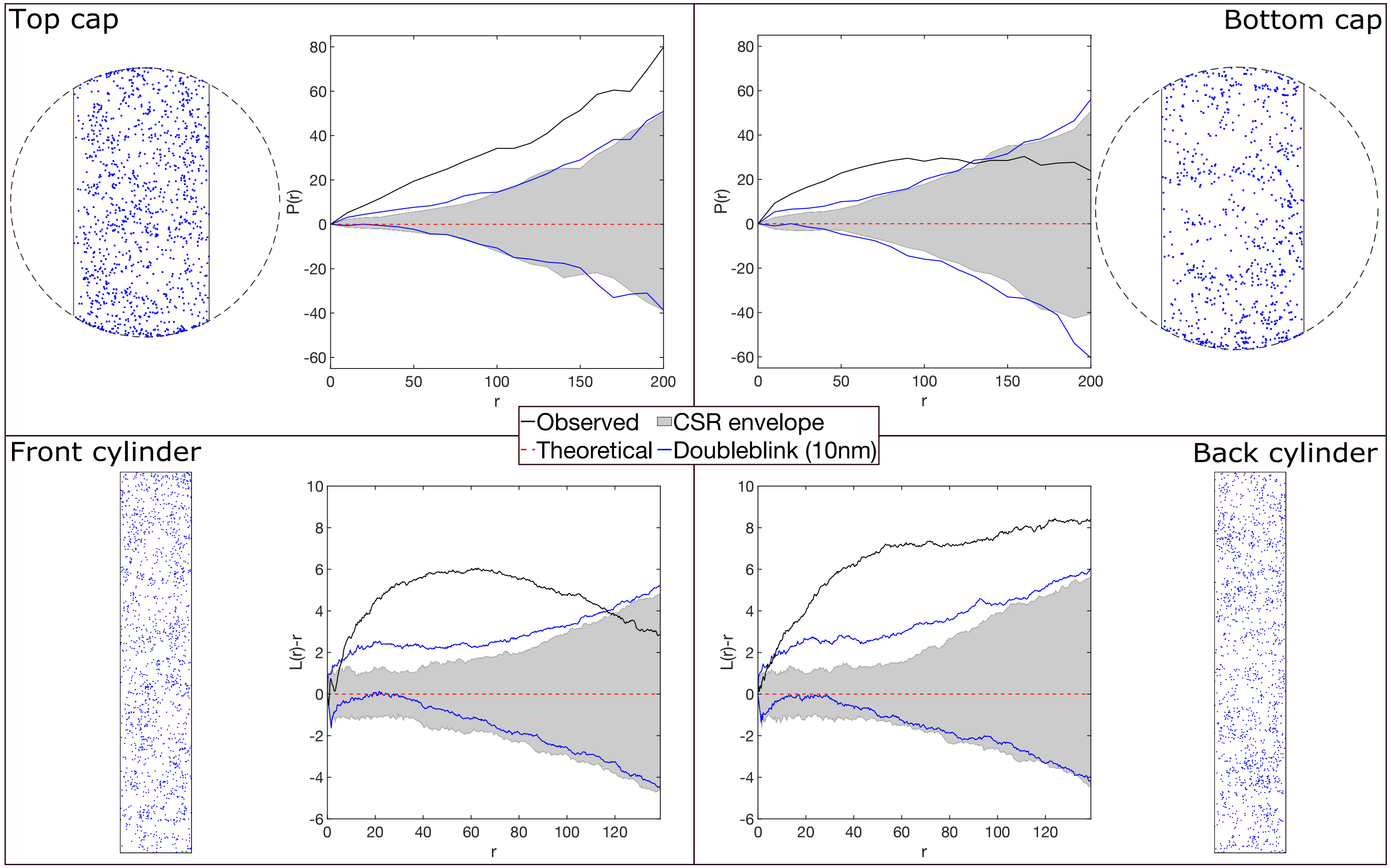

Supplementary Figure 9: **(Exponential)** Example piecewise analysis: *top left* - the observed homogeneous P-function on the top cap with a plot of the point pattern on the top cap, *top right* - the observed homogeneous P-function on the bottom cap with a plot of the point pattern on the bottom cap, *bottom left* - the observed homogeneous L-function on the front cylinder a plot of the point pattern on the front cylinder, *bottom right* - the observed homogeneous L-function on the back cylinder a plot of the point pattern on the back cylinder. Observed functional summary statistics is the black line, the theoretical value is the dashed red line and simulation envelope for the CSR process is grey and the simulation envelope for the DBM is given by the blue lines.

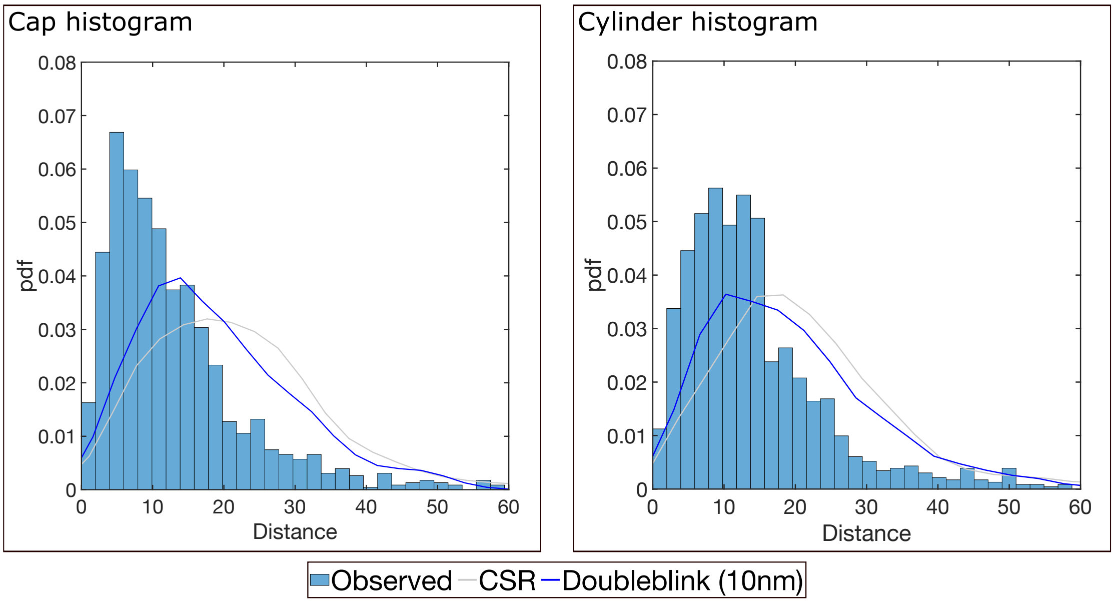

Supplementary Figure 10: **(Stationary)** Example histograms: *left* - nearest neighbour histogram for points on the spherical caps of the pill, *right* - nearest neighbour histogram for points on the cylinder of the pill. Grey line is the kernel estimate of the nearest neighbour distribution of a CSR process. Blue line is the kernel estimate of the nearest neighbour distribution of a DBM process.

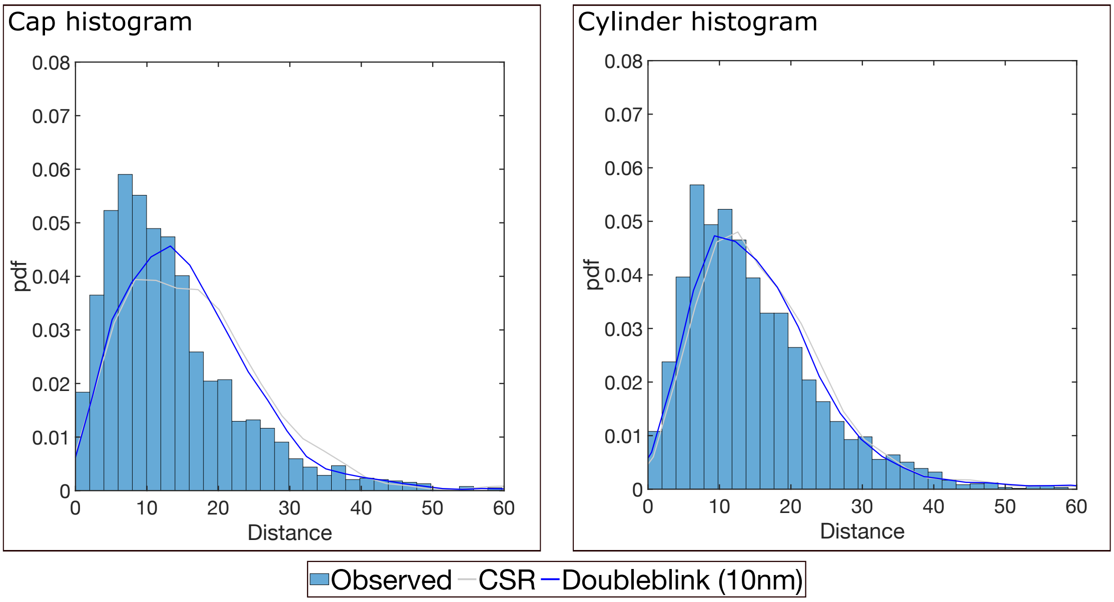

Supplementary Figure 11: **(Exponential)** Example histograms: *left* - nearest neighbour histogram for points on the spherical caps of the pill, *right* - nearest neighbour histogram for points on the cylinder of the pill. Grey line is the kernel estimate of the nearest neighbour distribution of a CSR process. Blue line is the kernel estimate of the nearest neighbour distribution of a DBM process.

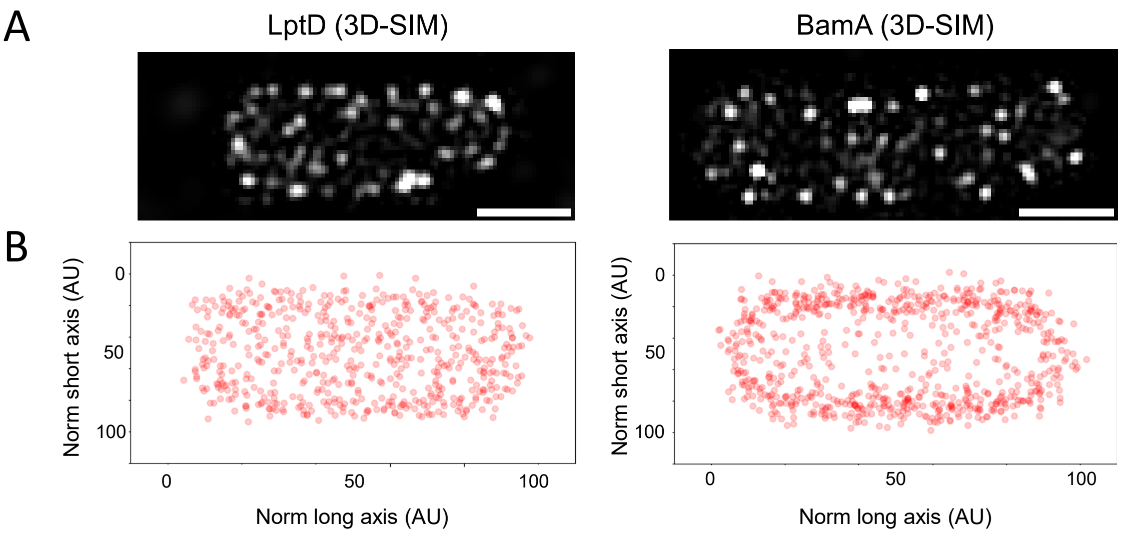

Supplementary Figure 12. BamA and LptD are similarly clustered in the *E. coli* OM.
E. coli cells were grown to mid-log phase, fixed and labelled using either αLptD or αBamA monoclonal antibodies conjugated to AF488. BamA was labelled as in (21), LptD as described in Materials and Methods. **(A)** Representative 3D-SIM images of LptD labelled cells compared to BamA labelled cells. The images demonstrate the clustering of both the Lpt and BAM biogenesis machineries over the bacterial cell surface. Scale bars, 1µm. **(B)** Integrated localization data from multiple cells showing LptD (n=583) and BamA (n=798) clusters are distributed through the *E. coli* OM. The long and short axes of the cells were normalized for the integrated analysis.

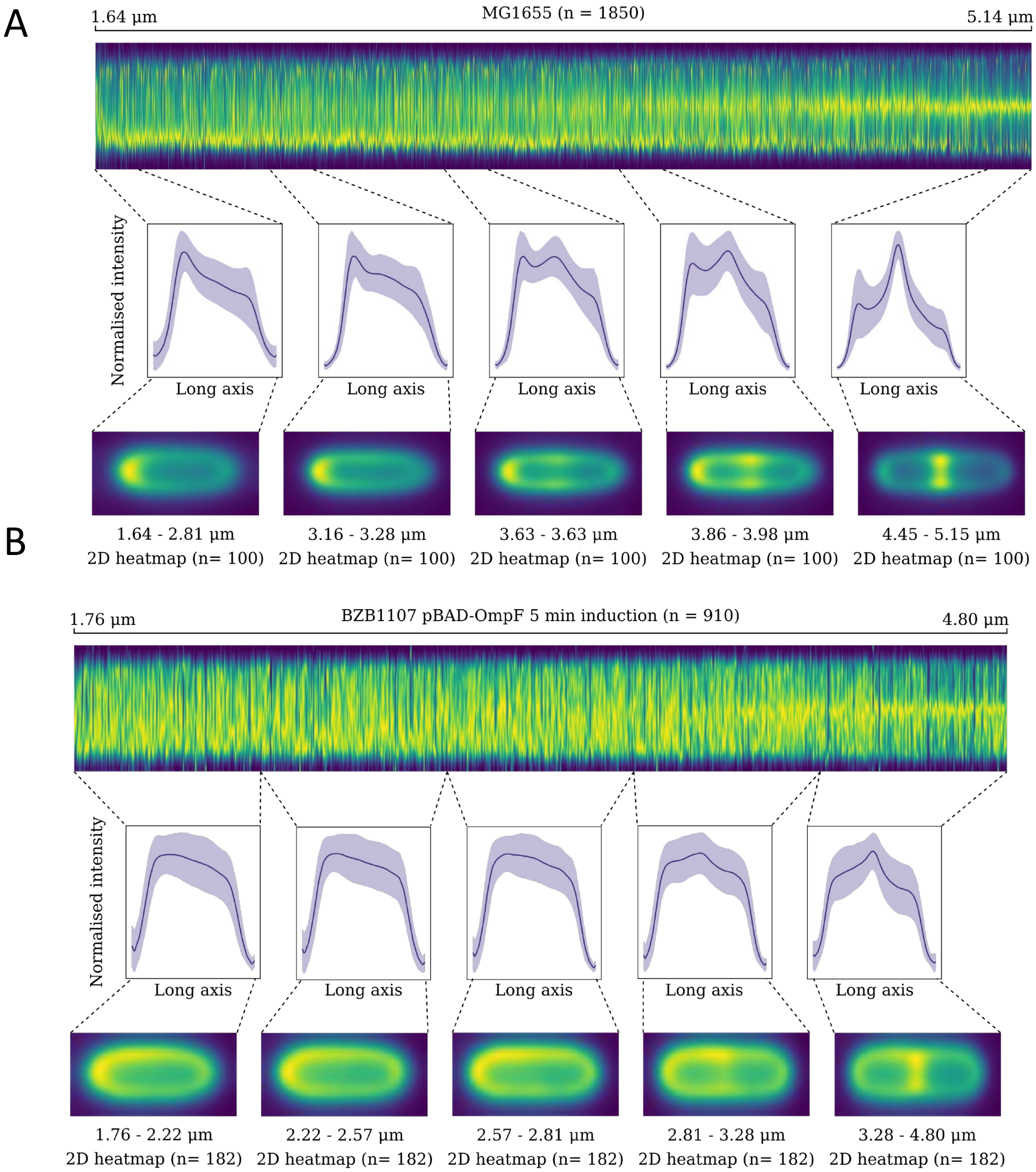

Supplementary Figure 13. The emergence of OmpF on the surface of *E. coli* is linked to the cell cycle. (**A**) OmpF distribution in *E. coli* MG1655 as a function of cell length. *Upper panel*, Kymograph wherein each vertical line of pixels represents the fluorescence intensity profile along the long axis of single cells. 1850 cells were used to construct the kymograph with lengths ranging from 1.64 to 5.14 µm. *Middle panels*, Average 1D long axis fluorescence intensity profiles extracted from 100 cell slices of the kymograph. *Dark purple line*, mean intensity profile; *light purple envelope*, (±) standard deviation. *Bottom panels*, Average 2D fluorescence intensity profiles from 100 cell kymograph slices, with length ranges denoted. (B) OmpF distribution in *E. coli* BZB1170 *ompF ompC* cells following a 5-minute induction of plasmid-encoded OmpF. Upper panel, Kymograph where cells were sorted by long axis length. 910 cells were used to construct the kymograph with lengths ranging from 1.76 to 4.80 µm. *Middle panels*, Average 1D long axis fluorescence intensity profiles extracted from 182 cell slices of the kymograph. *Dark purple line*, mean intensity profile; *light purple envelope*, (±) standard deviation. *Bottom panels*, Average 2D fluorescence intensity profiles from 182 cell kymograph slices, with length ranges denoted. In both native and plasmid expression, OmpF appearance in the OM is cell cycle dependent.

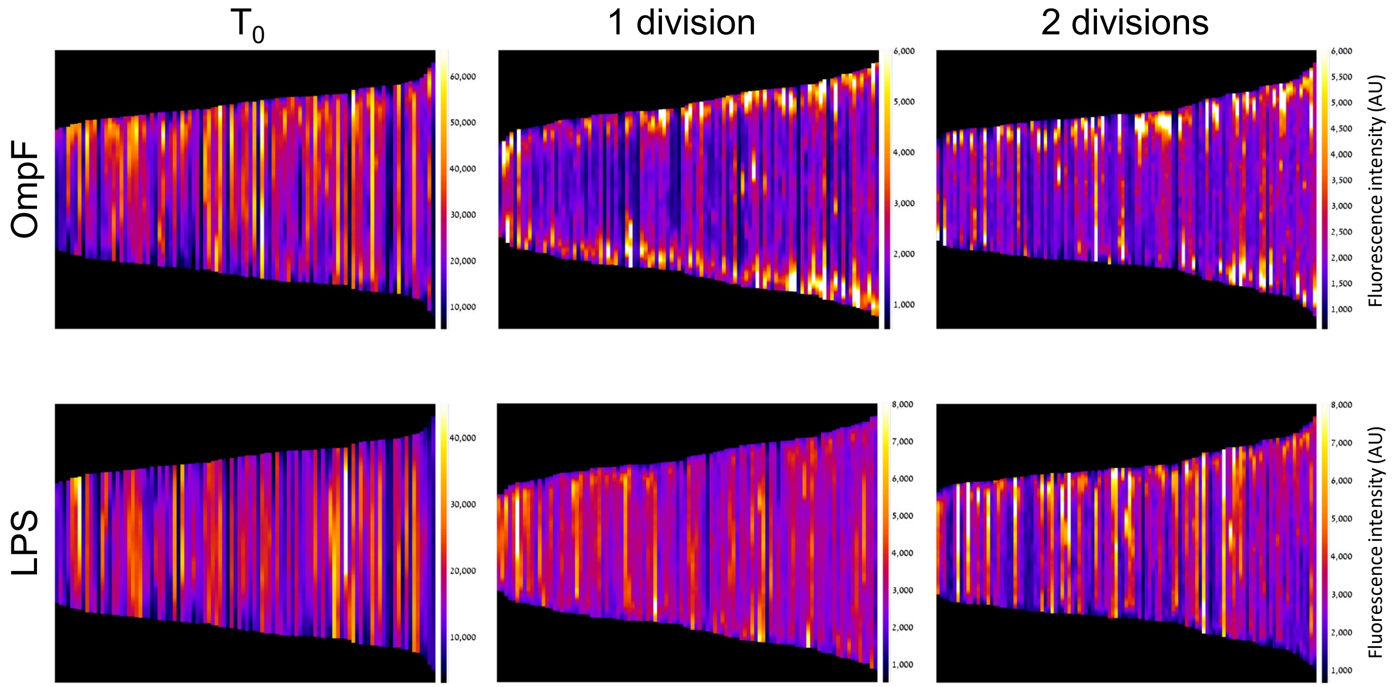

Supplementary Figure 14. OmpF and LPS exhibit fundamentally different distribution patterns as a result of cell growth

E. coli cells were labelled in stationary phase using KDO-azide and Alexa Fluor-555 for LPS and ColN-GFP for OmpF, respectively (see Materials and Methods). Cells were subsequently revived in fresh LB and imaged by TIRFM on agar pads at the indicated time points. Division times were calculated based on the OD_{600} of the culture following the resuspension in fresh media. For each time point, demographs of OmpF and LPS fluorescence intensity across the long axis is shown. OmpF and LPS profiles were analyzed on the same cells (n=100 for each time point).

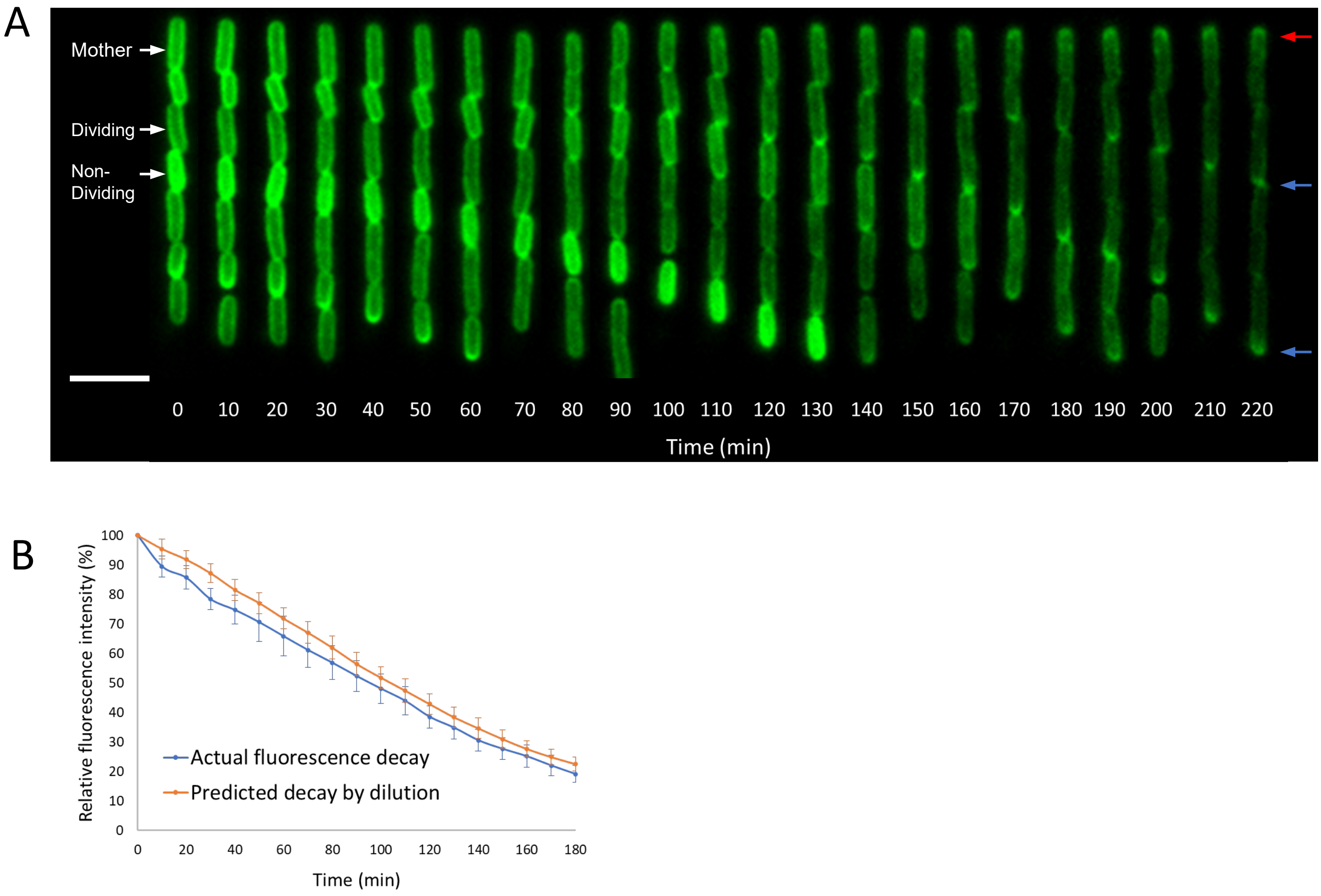

Supplementary Figure 15. Redistribution of surface LPS during cell growth.

LPS dilution and distribution in microfluidic channels for *E. coli* cells pulse-labelled with KDO-azide and Alexa Fluor- 488 sDIBO. **(A)** Time-lapse image of a single channel at the indicated time points post labelling. Cells representing dividing, nondividing and mother cell populations are indicated. Red arrow indicates retention of old LPS at the pole of the mother cell. Blue arrows indicate retention of old LPS at the poles of cells further up the growth channel. Scale bar, 5µm. **(B)** Decay of the relative fluorescence intensity in dividing cells compared to that predicted by simple dilution according to cell growth. The graph indicates that a model assuming simple dilution of old LPS is similar to that observed in the flow-channel experiments. Error bars represent the standard deviation for each group and time point. n= 47.

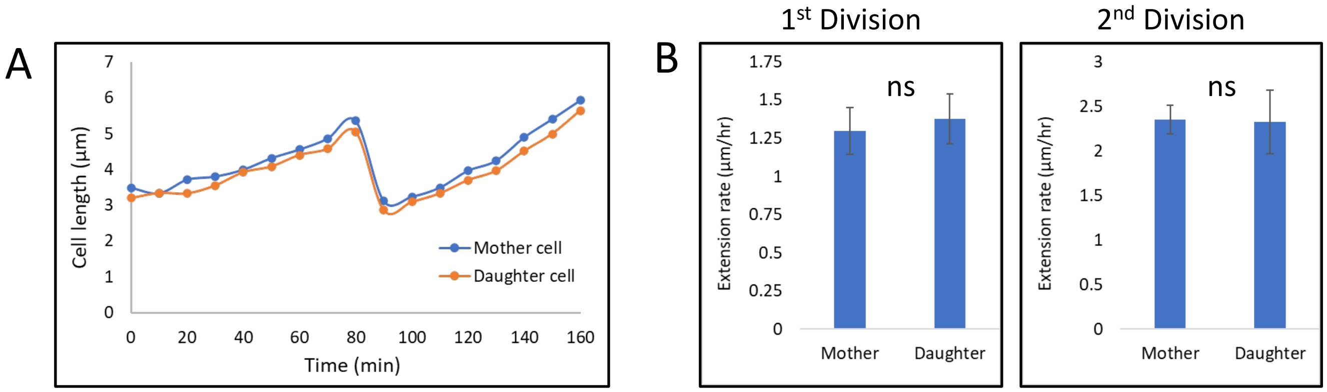

Supplementary Figure 16. Mother and daughter cells grow at a similar rate

Continuous tracking of cell length in microfluidic flow channels (Mother Machine) for growing E. coli cells pulse-labelled with KDO-azide and Alexa Fluor-488 sDIBO. See Figure 4. **(A)** A representative cell length comparison between mother and daughter cells throughout the time lapse experiment shown in Figure 4 and Movie S2. **(B)** Comparison of the calculated extension rate of mother vs daughter cells during the first and second cell divisions following LPS labelling. n=10.

Supplementary Movies legends

Movie 1. Super-resolution microscopy reveals the surface distribution of OmpF

Super-resolution reconstruction of OmpF organisation in E. coli MG1655 imaged by PALM. Shown are whole cell distribution followed by increased magnifications of specific OM regions highlighting OmpF clustering.

Movie 2. Tracking LPS labelling during cell growth in the Mother Machine setup

Continuous tracking of LPS distribution in microfluidic flow channels (Mother Machine) for growing *E. coli* cells pulse-labelled with KDO-azide and Alexa Fluor-488 sDIBO. Shown is a time-lapse movie of a single field of view containing 20 growth channels. Frames were taken at 10min intervals.

Movie 3. Tracking general cell growth in the Mother Machine setup

Continuous tracking of cell growth in microfluidic flow channels (Mother Machine) for *E. coli* cells pulse-labelled with KDO-azide and Alexa Fluor-488 sDIBO. Shown is a time-lapse movie of the epifluorescence channel allowing better comparative tracking of cell elongation and division. The presented field of view and imaging intervals are identical to Movie 2.

Supplementary Table 1. List of strains used in this study

**Strain**	**Description**	**Source**
BL21 (DE3)	*fhuA2 [lon] ompT gal (λ DE3) [dcm] ∆hsdS*<br>*λ DE3 = λ sBamHIo ∆EcoRI-B int::(lacI::PlacUV5::T7 gene1) i21 ∆nin5*	NEB
BW25113	∆(araD-araB)567 ∆lacZ4787(::rrnB-3) λ- rph-1<br>∆(rhaD-rhaB)568 hsdR514	Ref. (62)
MG1655	*λ rph-1*	Ref. (63)
BE3000	*∆ompC*	Ref. (64)
BZB1107	*∆ompF ∆ompC ∆lamB*	Ref. (65)
AB1157		Ref. (66)
GNE49	BW25113 *waaD*::Kan	Ref. (67)

Supplementary Table 2. List of plasmids used in this study

**Plasmid**	**Description**	**Source**
pKBJ51	Colicin N$^{1-185}$mCherry	Ref. (35)
pPGI1	Colicin N$^{1-185}$GFP	This study
pPGI3	Colicin N$^{1-185}$PAmCherry	This study
pNGH71	pBAD-HismycB OmpF	Ref. (68)
pNP4	GFP	Ref. (69)
pROD85	PAmCherry	Ref. (70)

Supplementary Table 3. List of primers used in this study

Primer	Description
5’-TTCCCCTCTAGATTTAAGAAGGAGAGGATCCTATGAGTAAAGGAGAAGAACTTTTCACTGGAGTTGTCCCA-3’	Amplify GFP from pNP4 and introduce 5’ BamHI site for pPGI1 (forward)
5’-GGATCCGCGACCCATTTGCTGTCCACCAGTCATCTCGAGTTTGTATAGTTCATCCATGCCATGTGTAATC-3’	Amplify GFP from pNP4 and introduce 3’ XhoI site for pPGI1 (reverse)
5’-CCGCTGCTGGTTCTGGGGATCCCGTGAGCAAGGGCGGCGAGG-3’	Amplify PAmCherry from pROD85 for pPGI3 (forward)
5’-CGAAGCAGCTCCAGCCTACACCCCTCGAGCTTGTACAGCTCGTCCATGCC-3’	Amplify PAmCherry from pROD85 for pPGI3 (reverse)

Supplementary Table 4. List antibodies and bacteriocins used in this study

Antibody/Colicin	Description	Dilution	Reference
αBamA-MAB2-AF488	Fab, Monoclonal	200nM	(21)
αlptD-AF488	Fab, Monoclonal	200nM	(24)
Colicin N^{1-185}mCherry	Engineered colicin	200nM	(24)
Colicin N^{1-185}GFP	Engineered colicin	200nM	This Study
Colicin N^{1-185}PAmCherry	Engineered colicin	200nM	This study

Supplementary References

(54) Jesper Moller and Rasmus Plenge Waagepetersen. Statistical inference and simulation for spatial point processes. CRC Press, 2003.

(55) Brian D Ripley. Modelling spatial patterns. Journal of the Royal Statistical Society: Series B (Methodological), 39(2):172-192, 1977.

(56) Scott Ward, Edward A. K. Cohen, and Niall Adams. Testing for complete spatial randomness on three dimensional bounded convex shapes. Spatial Statistics, 41:100489, 2021.

(57) Thomas Joseph Lawrence, Adrian Baddeley, Robin K Milne, and Gopalan Nair. Point pattern analysis on a region of a sphere. Stat, 5(1):144-157, 2016.

(58) Jesper M_ller and Ege Rubak. Functional summary statistics for point processes on the sphere with an application to determinantal point processes. Spatial Statistics, 18:4-23, 2016.

(59) Thomas Joseph Lawrence. Point pattern analysis on a sphere. Master's thesis, The University of Western Australia, 2018.

(60) Scott M Robeson, Ao Li, and Chunfeng Huang. Point-pattern analysis on the sphere. Spatial Statistics, 10:76-86, 2014.

(61) Adrian W Bowman and Adelchi Azzalini. Applied smoothing techniques for data analysis. Oxford Univeristy Press, New York, 1997.

(62) K. A. Datsenko, B. L. Wanner, One-step inactivation of chromosomal genes in Escherichia coli K 12 using PCR products. *Proceedings of the National Academy of Sciences of the United States of America* **97**, 6640-6645 (2000).

(63) M. S. Guyer, R. R. Reed, J. A. Steitz, K. B. Low, Identification of a sex-factor-affinity site in E. coli as gamma delta. *Cold Spring Harb Symp Quant Biol* **45 Pt 1**, 135-140 (1981).

(64) R. M. Garavito, J. P. Rosenbusch, Isolation and crystallization of bacterial porin. *Methods Enzymol* **125**, 309-328 (1986).

(65) R. Ghosh, M. Steiert, A. Hardmeyer, Y. F. Wang, J. P. Rosenbusch, Overexpression of outer membrane porins in E. coli using pBluescript-derived vectors. *Gene Expr* **7**, 149-161 (1998).

(66) S. K. Dewitt, E. A. Adelberg, The Occurrence of a Genetic Transposition in a Strain of Escherichia Coli. *Genetics* **47**, 577-585 (1962).

(67) T. Baba *et al.*, Construction of Escherichia coli K-12 in-frame, single-gene knockout mutants: the Keio collection. *Mol Syst Biol* **2**, 2006 0008 (2006).

(68) S. A. Ionescu *et al.*, Orientation of the OmpF Porin in Planar Lipid Bilayers. *Chembiochem : a European journal of chemical biology* **18**, 554-562 (2017).

(69) M. A. Gerding, Y. Ogata, N. D. Pecora, H. Niki, P. A. de Boer, The trans-envelope Tol-Pal complex is part of the cell division machinery and required for proper outer-membrane invagination during cell constriction in E. coli. *Molecular microbiology* **63**, 1008-1025 (2007).

(70) S. Uphoff, R. Reyes-Lamothe, F. Garza de Leon, D. J. Sherratt, A. N. Kapanidis, Single-molecule DNA repair in live bacteria. *Proceedings of the National Academy of Sciences of the United States of America* **110**, 8063-8068 (2013).
